# Evolution of intra-tumoral heterogeneity across different pathological stages in papillary thyroid carcinoma

**DOI:** 10.1186/s12935-022-02680-1

**Published:** 2022-08-22

**Authors:** Ornella Affinito, Francesca Maria Orlandella, Neila Luciano, Marco Salvatore, Giuliana Salvatore, Monica Franzese

**Affiliations:** 1 IRCCS Synlab SDN S.P.A., Via Gianturco 113, 80143 Naples, Italy; 2 Dipartimento di Scienze Motorie e del Benessere, Università Degli Studi di Napoli Parthenope, Naples, Italy; 3 CEINGE - Biotecnologie Avanzate S.C.a.R.L., Naples, Italy; 4 Dipartimento di Scienze Biomediche Avanzate, Università Degli Studi di Napoli “Federico II”, Naples, Italy; 5 Bio Check Up S.R.L, Naples, Italy

**Keywords:** Papillary thyroid cancer, Tumor heterogeneity, TCGA-THCA, Tumor stages, Mutational signatures, Prognosis

## Abstract

**Background:**

Intra-tumor heterogeneity (ITH) results from the continuous accumulation of mutations during disease progression**,** thus impacting patients’ clinical outcome. How the ITH evolves across papillary thyroid carcinoma (PTC) different tumor stages is lacking.

**Methods:**

We used the whole-exome sequencing data from The Cancer Genome Atlas Thyroid Cancer (TCGA-THCA) cohort to track the ITH and assessed its relationship with clinical features through different stages of the PTC progression. We further assayed the expression levels of the specific genes in papillary thyroid cancer cell lines compared to an immortalized normal thyroid epithelial cell line by qRT-PCR.

**Results:**

We revealed the timing of mutational processes and the dynamics of the temporal acquisition of somatic events during the lifetime of the PTC. ITH significantly influences the PTC patient’s survival rate and, as genetic heterogeneity increases, the prognosis gets worse in advanced tumor stages. ITH also affects the mutational architecture of each clinical stage which is subject to periodic fluctuations. Different mutational processes may cooperate to shape a stage-specific mutational spectrum during the progression from early to advanced tumor stages. Moreover, different evolutionary paths characterize PTC progression across pathological stages due to both mutations recurrently occurring in all stages in hotspot positions and distinct codon changes dominating in different stages. A different expression level of specific genes also exists in different thyroid cancer cell lines.

**Conclusions:**

Our findings suggest ITH as a potential unfavorable prognostic factor in PTC and highlight the dynamic changes in different clinical stages of PTC, providing some clues for the precision medicine and suggesting different diagnostic decisions depending on the clinical stages of patients. Finally, complete clear guidelines to define risk stratification of PTC patients are lacking; thus, this work could contribute to defining patients who need more aggressive treatments and, in turn, could reduce the social burden of this cancer.

**Supplementary Information:**

The online version contains supplementary material available at 10.1186/s12935-022-02680-1.

## Background

Tumorigenesis is often initiated by a single mutated neoplastic cell evolving through a series of sequential clonal or subclonal mutational events, thus influencing the course of disease [1, 2]. In this way, cell clones diversify, resulting in intratumor heterogeneity (ITH) [3, 4]. As a result, cancer evolves as a mosaic entity composed of a mixture of cells with distinct genetic, phenotypic or behavioral features within the same tumor [5]. Changes in selection pressure within tumor microenvironment may also enhance clonal diversity or allow the positive selection of more aggressive clones with subclonal mutations that increase the tumor’s fitness to its environment and determine a more aggressive evolution of diseases [6]. ITH may provide detailed records of tumor clonal evolution history and evolutionary dynamics of mutations, chronologically accumulated and selected during the lifetime of a tumor [7–9]. Deciphering and tracking the clonal evolution pattern could provide valuable information regarding crucial genetic events in tumorigenesis and progression, thus greatly benefiting therapy selection and prognosis management. Indeed, ITH represents a significant challenge in the implementation of precision medicine because it is responsible for the progression from an early tumor stage to a more aggressive cancer. Moreover, ITH is one of the major causes of poor prognosis, treatment failure and drug resistance [8, 10–12]. Some evidence suggests that ITH influences the clinical outcome in several cancers, such as chronic lymphocytic leukemia [6], head and neck cancer [10, 11], colorectal [12] and lung adenocarcinoma [13]. Moreover, ITH has the potential to be a valuable predictor for clinical outcomes [14] and may serve as a clinically useful biomarker in the development of personalized therapies and clinical outcomes [15, 16]. Thus, exploring intratumor heterogeneity and tumor evolution is of great clinical importance because, depending on the tumor evolution, different clinical approaches may exist in terms of diagnosis, prognosis, and treatment of the individuals [17].

Despite its potential clinical relevance, ITH was poorly investigated in papillary thyroid carcinoma (PTC). PTC is the most frequent endocrine tumor accounting for almost 80% of thyroid cancer cases. Somatic mutations of genes involved in the mitogen-activated protein kinase (MAPK) signaling pathway, including point mutations in *BRAF*, *RAS* genes [18–21] and *RET/PTC* rearrangement [22]. Although PTC has in general a good prognosis with a 5-year survival of over 95% [23], in about 85% of case subjects, a small fraction (10–20%) shows higher aggressiveness with either local or distant relapse [24, 25]. A large Cancer Genome Atlas (TCGA) Research Network study [26], based on genetic, epigenetic and transcriptomic analysis of almost 500 PTCs, demonstrated substantial inter-tumor heterogeneity in PTCs concerning their molecular alterations. Moreover, they found that driver mutations in genes such as *BRAF*, *NRAS*, *HRAS*, *KRAS*, and *EIF1AX* were present in most PTC cases and were largely clonal. On the other side, as regards the intra-tumoral heterogeneity, most studies focused on the most prevalent genetic alterations in PTC, the *BRAF* V600E mutation and *RET* rearrangements [27–30]. However, while each cancer cell may contain a driver mutation, the PTC cells are still proliferating and can diversify through the gain of subclonal genetic alterations that can be positively selected and might impact prognosis and clinical outcome [31]. Moreover, these non-driver alterations may provide insight into a tumor’s history and help to identify mutational processes occurring during tumorigenesis [32]. Some evidence supports the occurrence of ITH in PTC [33], but the relevance of subclonality in PTC is still debated [34]. However, a comprehensive analysis taking into account the changes and the impact of ITH through different pathological stages of the PTC, along with the timing of mutational processes and the dynamics of the temporal acquisition of somatic events, is lacking.

In the current study, we analyzed the whole-exome sequencing data from The Cancer Genome Atlas Thyroid Cancer (TCGA-THCA) cohort to efficiently and comprehensively evaluate somatic variants across clinical stages of PTC. We tracked the intra-tumoral genetic heterogeneity by mutant-allele tumor heterogeneity (MATH) algorithm [35] and assessed its relationship with clinical features through different stages associated with the PTC progression. We also revealed how mutational processes vary over time during tumor evolution and identified several evolutionary patterns depending on the behavior of the mutated genes. We further investigated the relationship between the mutational status and RNA expression level. These findings highlighted the dynamic changes of oncogenesis in different clinical stages of PTC, providing some clues for the development of precision medicine and the improvement of diagnostic strategies in PTC patients.

## Methods

### Data source

Publicly available data were obtained from The Cancer Genome Atlas (TCGA) Research Network: https://www.cancer.gov/tcga. Mutation annotation format (MAF) files for single nucleotide variants (SNVs) analyzed with VarScan2 variant Aggregation and Masking workflow were downloaded by using TCGAbioloinks R/Bioconductor package [36]. Survival and clinical data were downloaded from the cancer genomics cBioPortal (http://www.cbioportal.org/) [37].

There were 487 patients with mutation data and 507 with available clinical and survival data. Data were filtered to exclude patients without mutation or clinical information. We also excluded patients without available information on tumor stage and retained patients with the following histological types: classical, follicular and tall cell variant papillary thyroid carcinoma. The final dataset was composed of 474 patients consisting of 265 stage 1, 50 stage 2, 108 stage 3 and 51 stage 4.

### Somatic mutation analysis and measurement of heterogeneity

We used the R/Bioconductor package Maftools [38] to efficiently and comprehensively analyze somatic variants in PTC. Genetic intra-tumor heterogeneity (ITH) was assessed by mutant-allele tumor heterogeneity (MATH) score [35]. To estimate MATH scores, the *InferHeterogeneity* function of the Maftools package was used, which infer clonality by clustering variants with similar allele frequencies and removing patients with less than 3 variants*.* The MATH score for each PTC patient was calculated according to the method described by Mroz et al. [10]. The MATH score is defined as the percentage ratio of the median absolute deviation (MAD) and the median of the distribution of MAFs among the tumor’s mutated genomic loci: MATH = 100*MAD/median.

For each tumor stage, patients were categorized according to tumor heterogeneity into high- and low-MATH groups by the median of the MATH scores. High- and low-MATH groups were then analyzed with respect to clinicopathologic features and Progression-free survival (PFS) data.

### Mutation signature analysis

Somatic mutations of each sample were classified according to base substitution (C > A, C > G, C > T, T > A, T > C, T > G) and immediately 5′/3′ base information. We identified mutational context based on the human reference genome hg38 through *BSgenome.Hsapiens.UCSC.hg38* R package. The resulting triplet SNV spectra were analyzed for contributions of known mutational signatures in Catalogue of Somatic Mutations in Cancer (COSMIC; https://cancer.sanger.ac.uk/cosmic/signatures). Mutational signatures were predicted using the deconstructSigs R package [39]. This tool evaluates the contribution of the signatures reported in COSMIC (used as reference mutational signatures) to the mutational profile of the somatic SNVs in each tumor stage. Mutations not included in previously identified signatures were classified as “unknown”.

### Expression level analysis using web resources

The expression level of the common mutated genes (*JMJD1C*, *MALAT1*, *MUC16*, *PDZD2*, *PKHD1*, *RYR1*, *SLA* and *TTN*) was analyzed across thyroid tumor tissues and normal thyroid samples and across thyroid cancer stages deposited in TCGA project using UALCAN data portal (http://ualcan.path.uab.edu) [40].

### RNA extraction and qRT-PCR

Total RNA was extracted from an immortalized normal thyroid epithelial cell line (Nthy-ori 3-1) and from three papillary thyroid cancer cell lines (K1, TPC-1 and BCPAP) using TRIzol reagent (Catalog number 15596026) purchased from Thermo Fisher Scientific (Waltham, USA).

After extraction, RNA was quantified using the NanoDrop spectrophotometer (Thermo Fisher Scientific). Next, cDNA was synthesized using 1 µg of total RNA from each sample using QuantiTect Reverse Transcription (Catalog number 205311) purchased from Qiagen (Hilden, Germania) according to the manufacturer’s instructions.

The qRT-PCR was performed using iQ™ SYBR Green Supermix (Catalog number1708880) purchased from BioRad (Hercules, United States). The thermal cycler conditions were: 1:30 min at 95 °C for the denaturation and enzyme activation, followed by 40 cycles of 20 s at 95 °C for denaturing and 1 min at 60 °C for annealing/extension, according to the manufacturer's instructions. Primers used were:

MUC16: reverse 5′-caacctcacctcctcccatt-3′; forward 5′-atctgaagtgtggctcagct-3′;

JMJD1C: reverse 5′-gcctccaactctaatacccga-3′; forward 5′ atggacgcacaatgacagatg-3′;

PDZD2: reverse 5′-gacttccaatcgagtgactgc-3′; forward 5′ cagcagctcatctcctaagga-3′;

RYR1: reverse 5′-gcgctgttggaagtactactg-3′; forward 5′-tcaaagatgccccagaagagt-3′;

SLA: reverse 5′-cttgccgtgctaagtgactac-3′; forward 5′-accgacagtgagtaaaaccct-3′;

β-ACTIN: reverse 5′-ccaaccgcgagaagatga-3′; forward 5′-ccagaggcgtacagggatag-3′.

The relative expression level (expressed as fold change) was determined by applying the formula 2^−ΔΔCt^ [41] where the Ct-value of each gene was technically normalized using β-ACTIN. The experiment was carried out three independent times in triplicate.

### DNA sequencing

Genomic DNA was purified from cultured thyroid cells (Nthy-ori 3-1, K1, TPC-1 and BCPAP) using QIAamp DNA mini kit (Catalog number, 51304) purchased from Qiagen (Hilden, Germany) and quantified with the Nanodrop spectrophotometer (Thermo Fisher Scientific, Massachusetts, USA). Next, we designed specific primers able to amplify the exonic region of genes containing the high impact mutations. PCR was carried out on Applied Biosystems 2720 Thermal Cycler (Thermo Fisher Scientific) using Taq DNA polymerase (Catalog Number EP0401, Thermo Fisher Scientific) with the following thermal cycles: denaturation at 95 °C (for 3 min), 40 cycles at 95 °C (for 30 s), 60 °C (for 30 s), 72 °C (for 1 min), followed by 72 °C (for 5 min).

The genes, the exons harboring the high impact mutations found in PTC patients, and the primer sequences used are listed in Additional file 1: Table S1.

The amplicons sequencing was performed as follows: 8 μl of primer forward (concentrated 2 μM) and 20 μl of DNA (concentrated 16 ng/μl). Sanger electropherograms were analyzed to evaluate the presence/absence of the indicated mutations.

### Statistical analysis

The normal distribution assumption for continuous variables was assessed by Shapiro–Wilk test. For comparisons between two groups, a two-tailed *t*-test for independent samples (for normally distributed data) or a Wilcoxon rank sum test (for not normally distributed data) was used. Comparisons among more than two groups were made using the Kruskal–Wallis test. p-values were also corrected for multiple testing by the Bonferroni method. Progression-free survival (PFS) data were used as endpoints for survival analysis. Survival rates were analyzed with the Kaplan–Meier method and the statistical relevance of the differences between survival curves was assessed by log-rank test. Univariate analysis Cox proportional hazards regression models were applied to evaluate the prognostic value of the MATH score and to analyze the effects of clinical confounding factors on survival. All confidence intervals (CIs) were stated at the 95% confidence level. Statistical significance was set at p-value ≤ 0.05. All analyses were performed using R 3.6.0 (https://www.r-project.org/).

## Results

### Tumor stage-related survival analysis

We analyzed exome sequencing data from 474 patients with PTC obtained from the TCGA data portal. Clinicopathological information of the patients included in the current analysis is summarized in Table 1 and Additional file 2: Table S2.

**Table 1:** Clinical and pathological characteristics of patients in TCGA papillary thyroid carcinoma cohort

Clinical features	Category	PTC, *n* = 474
Age	Median (range)	46 (15–89)
Gender	Female	351
	Male	123
AJCC Tumor stage	Stage 1	265
	Stage 2	50
	Stage 3	108
	Stage 4	51
AJCC T stage	T1	135
	T2	160
	T3	156
	T4	22
	TX	1
AJCC M stage	M0	267
	M1	9
	MX	198
AJCC N stage	N0	217
	N1	209
	NX	48
Primary neoplasm focus type	Unifocal	255
	Multifocal	210

For each clinical feature, patients labeled as “Not available” or “Unknown” are not shown

**Fig. 1:**
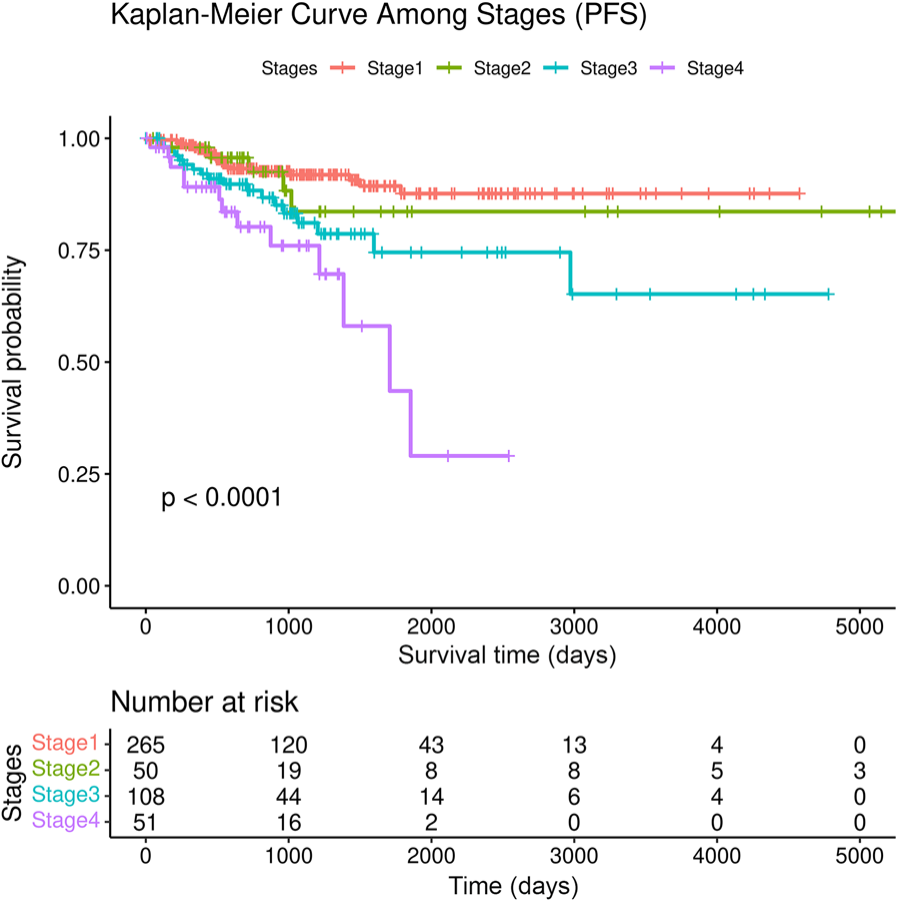
Kaplan–Meier analysis for Progression-Free survival rate among tumor stages. Log-rank test was performed to evaluate the survival differences

A Kaplan–Meier survival analysis was conducted using PFS data to analyze the survival probability in different tumor stages (Fig. 1). The results showed a statistically significant difference in survival probability depending on the tumor stage (Log-rank test; p-value < 0.0001), with a worse survival odd for patients in stage 4. In addition, we performed an univariate Cox proportional hazards regression to evaluate the association between different tumor stages and prognosis (Table 2). The analysis showed that stages 3 and 4 were significantly associated with a poor prognosis (p-value = 0.0073 and p-value = 1.9e-05, respectively). Instead, patients in stage 2 also had a poor survival but not significant (p-value = 0.61).

**Table 2:** Univariate Cox proportional hazards regression model

Tumor stage	HR	95%CI	p-value
Stage 2 vs stage 1	1.29	0.48–3.43	0.61
Stage 3 vs stage 1	2.39	1.27–4.53	0.0073**
Stage 4 vs stage 1	4.63	2.29–9.36	1.9e−05***

*HR* hazard ratio, *CI* confidence interval

*P < 0.05; **P < 0.01; ***P < 0.001

### Potential role of the MATH score in PTC for tumor stage-related survival

**Fig. 2:**
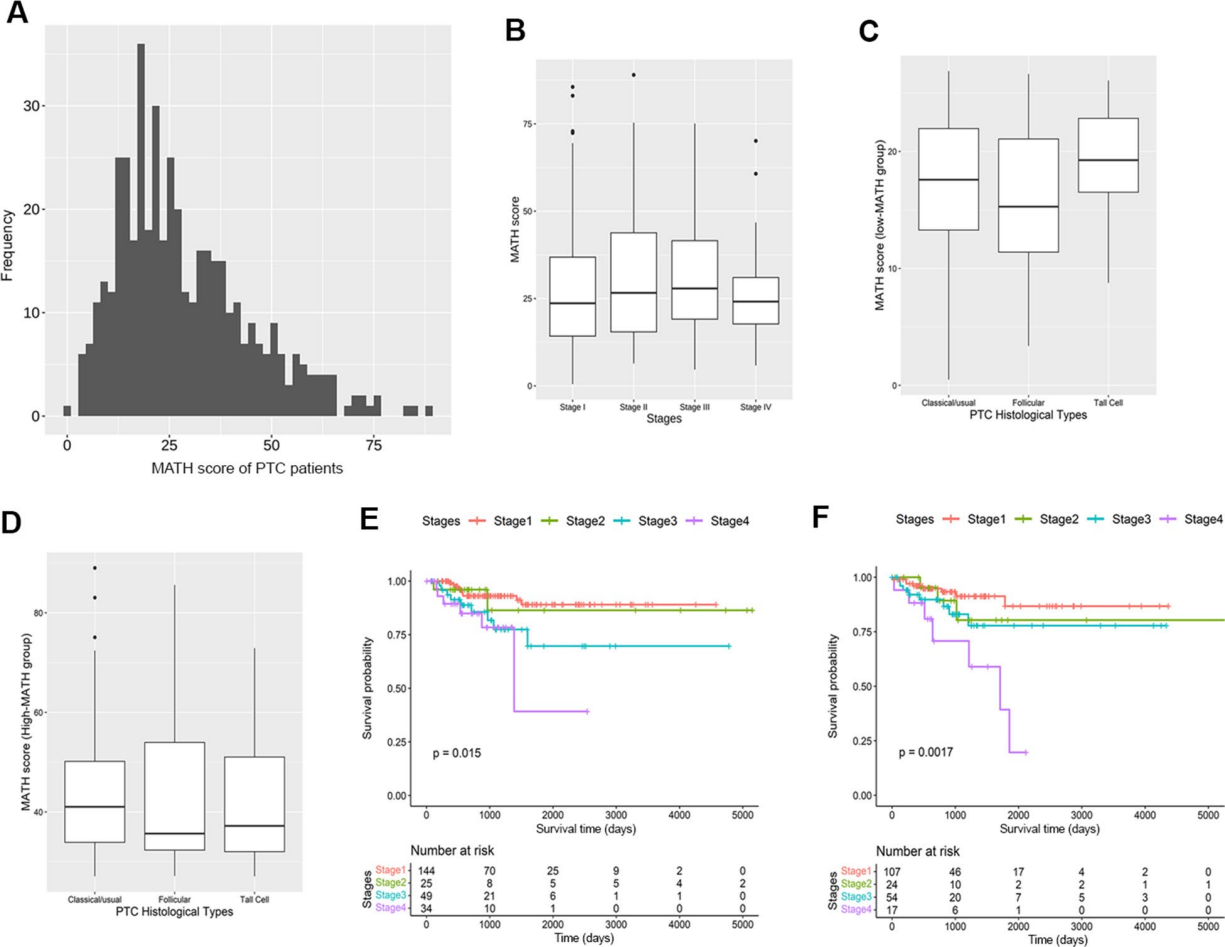
Frequency distribution of MATH scores among PTC patients. The MATH score represents a percentage measure of the level of intra-tumor heterogeneity and was calculated for each tumor as described in Material and Methods. **A** MATH values are displayed along the horizontal axis and the number of patients (frequency) with MATH within the specific ranges is displayed on the vertical axis. **B** Boxplots show the MATH scores distribution in different tumor stages of PTC. Boxplots show the MATH scores distribution in different PTC histological types (Classical/Usual, Follicular, Tall Cell) in **C** low-MATH and **D** high-MATH patients’ groups. **E** Kaplan–Meier survival curves in the low-MATH group according to tumor stage; **F** Kaplan–Meier survival curves in the high-MATH group according to tumor stage. *MATH* mutant allele tumor heterogeneity; *PTC* papillary thyroid carcinoma

We evaluated whether intratumor heterogeneity (ITH) could explain the different prognosis of tumor stages. ITH can be assessed using the mutant allele tumor heterogeneity (MATH) score. As reported in Fig. 2A, the MATH scores distribution showed a broad spectrum of values (from 0.49 to 89.01 with a mean ± standard deviation (SD) of 28.64 ± 16.33), suggesting that PTC patients exhibit remarkable intra-tumoral heterogeneity. MATH score distribution according to tumor stage highlighted a statistically significant difference (Kruskal–Wallis rank sum test; p-value = 0.033) (Fig. 2B). According to the median value (25.16) of the MATH score, patients were stratified into two groups: the low-MATH group (252 patients) and the high-MATH group (202 patients), with mean ± SD of 16.92 ± 6.15 and 43.26 ± 12.92, respectively. Any statistically significant difference (Kruskal–Wallis rank sum test; p-value > 0.05) was observed in MATH score distribution between different PTC histological types (classical/usual, follicular, tall cell) in either MATH group (Fig. 2C, D).

Next, we investigated the prognostic significance of MATH for each MATH group through a Kaplan–Meier survival analysis (Fig. 2E, F) and an univariate Cox proportional hazards regression (Table 3). Kaplan–Meier survival analysis showed that the MATH values were significantly associated with tumor stage (Fig. 2E, F). Specifically, we found that patients in advanced stages (stage 3 and stage 4) exhibited shorter PFS rates than those in early stages (stage 1 and stage 2) in both MATH groups (Log-rank test; p-value = 0.015 for the low-MATH group; p-value = 0.0017 for the high-MATH group) (Fig. 2E, F). The univariate Cox proportional hazards regression analysis underlined the role of MATH score as potential risk factor associated with PTC aggressiveness. Specifically, low MATH score showed a statistical significance in stage 3 (p-value = 0.021) and stage 4 (p-value = 0.007), whereas high MATH score showed a statistical significance in stage 4 (p-value = 0.0005) (Table 3).

**Table 3:** Univariate Cox proportional hazards regression model in low-MATH group and high-MATH group

Tumor stage	Low-MATH group			High-MATH group		
	HR	95% CI	P-value	HR	95% CI	P-value
Stage 2 vs Stage 1	1.23	0.27–5.62	0.790	1.69	0.45–6.36	0.441
Stage 3 vs Stage 1	2.89	1.17–7.12	0.021*	2.16	0.81–5.76	0.124
Stage 4 vs Stage 1	4.05	1.45–11.32	0.007**	6.15	2.22–s17.00	0.0005***

*HR* hazard ratio, *CI* confidence interval

^*^P < 0.05; **P < 0.01; ***P < 0.001

### Relationship between MATH and clinical features

We also investigated whether the tumor heterogeneity was associated with clinical features of PTC patients (Table 4, Additional file 3: Table S3 and Additional file 4: Table S4). We performed a Kaplan–Meier analysis followed by a univariate Cox regression model to establish the impact of different clinical features on survival and prognosis in low and high MATH groups. In the low-MATH group for stage 4, M stage was significantly associated with survival (Log-rank test; p-value = 0.02) with a poorer prognosis (p-value = 0.04) for M1 stage (p-value = 0.04) (Table 4). Moreover, the primary neoplasm focus type and neoplasm length clinical features were significantly associated with PFS (Log-rank test; p-value = 0.007 and p-value = 0.01, respectively), but their prognostic value was not significant (p-value > 0.05) (Table 4). All the other clinical features were not significantly associated with survival and prognosis in the low-MATH group (Additional file 3: Table S3). None of the clinical features was significantly associated with survival and prognosis in the high-MATH group (Additional file 4: Table S4).

**Table 4:** Relationship of clinical variables with progression free survival (PFS) by univariate Cox proportional hazards analysis in low-MATH group

Variables	Stage 1			Stage 2			Stage 3			Stage 4		
	P-value Log-rank test	Univariate Cox analysis		P-value Log-rank test	Univariate Cox analysis		P-value Log-rank test	Univariate Cox analysis		P-value Log-rank test	Univariate Cox analysis	
		HR (95% CI)	P-value Wald Test		HR (95% CI)	P-value Wald Test		HR (95% CI)	P-value Wald Test		HR (95% CI)	P-value Wald Test
M stage	0.7			0.07			0.6			0.02		
M1 vs M0		–	–		6.3 (0.56–70.91)	0.14		–	–		14.38 (1.12–185.41)	0.04
MX vs M0		1.26 (0.36–4.36)	0.71		0 (0–Inf)	1		0.68 (0.16–2.79)	0.59		2.14 (0.19–23.61)	0.54
Primary neoplasm focus type	0.8			0.9			0.9			0.007		
Unifocal vs multifocal		0.88 (0.25–3.12)	0.84		1.18 (0.11–13.12)	0.89		0.95 (0.25–3.6)	0.93		NA	NA
Neoplasm length	0.6			0.07			0.4			0.01		
High vs low		1.53 (0.36–6.41)	0.56		0 (0–Inf)	1		1.91 (0.47–7.71)	0.36		NA	NA

Statistical significance of differences between Kaplan–Meier survival curves was assessed by Log-rank test. Statistical relevance as prognostic value was assessed by Wald test

### Exploring the PTC intra-tumoral heterogeneity

**Fig. 3:**
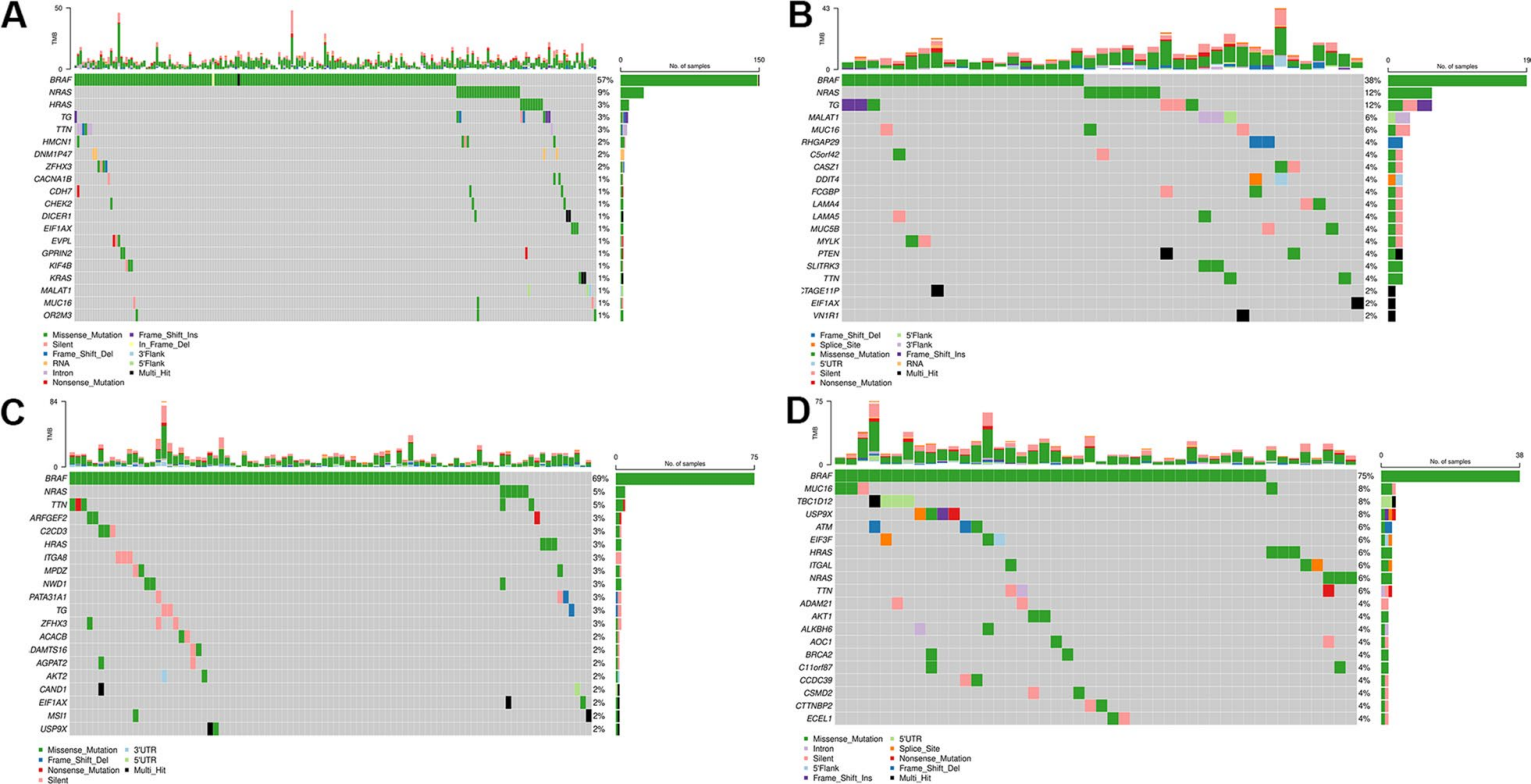
Oncoplots of the most frequently mutated genes in different PTC stages. Oncoplots display the most frequently mutated genes in **A** stage 1, **B** stage 2, **C** stage 3, and **D** stage 4. Each column represents a sample and each row a different gene. Genes are ordered by their mutation frequency. The top barplot shows the frequency of mutations for each patient, while the right barplot shows the frequency of mutations in each gene. Colors indicate different mutation types (see legend for details)

To better understand the intratumor heterogeneity of PTC, we explored the mutational landscape in different tumor stages. We calculated the mutation rates of the top 20 mutated genes for each stage and identified numerous somatic mutations. We identified several somatic mutations that frequently occur in PTC, along with the most common and well-described [18–21] with particularly high frequencies (Fig. 3A–D; Additional file 5: Table S5). Indeed, the serine/threonine kinase *BRAF* gene presented relatively high mutation rates in all stages (57% in stage 1, 38% in stage 2, 69% in stage 3, 75% in stage 4). The most frequent mutation was the well-known hotspot *BRAF* c.1799 T > A (p.V600E). In addition, in stage 1 one patient had the simultaneous occurrence of two *BRAF* mutations at positions c.1799 T > A (p.V600E) and c.1800G > A (p.V600V). Also, one patient had the *BRAF* c.1801A > G (p.K601E) mutation, while another one had the *BRAF* c.1467_1481delACCTACACCTCAGCA (p.P490_Q494del) deletion. In stage 2, one patient had the *BRAF* c.1801A > G (p.K601E) mutation.

The second most mutated gene in stage 1 was the GTPase *NRAS* (9%), with the c.182A > G (p.Q61R) and c.181C > A (p.Q61K) mutations. In stage 2 the second most mutated genes were the GTPase *NRAS* (12%), with the same mutations of the stage 1, and the Thyroglobulin (*TG*) (12%), with the c.1038dupG (p.H347Afs*14), c.1663G > A (p.E555K), c.450G > T (p.G150G), c.490 T > G (p.C164G), c.5707_5708dupTT (p.C1904Sfs*7), c.666 T > C (p.S222S) mutations. In stage 3, the second most mutated genes were the GTPase *NRAS* (5%), with the same mutations of the stage 1, and Titin (*TTN*) (5%), with the c.24810G > C (p.E8270D), c.68948C > T (p.T22983I), c.83902C > T (p.R27968*), c.97262 T > C (p.M32421T), c.9769C > T (p.R3257C). In stage 4, the second most frequent mutated genes were Mucin 16, Cell Surface Associated (*MUC16*) (8%), with the c.1295C > A (p.T432N), c.18018C > A (p.H6006Q), c.22026A > C (p.T7342T), c.32174C > G (p.P10725R) mutations; TBC1 Domain Family Member 12 (*TBC1D12*) (8%), with the c.-1G > A and c.-3C > T mutations; and Ubiquitin Specific Peptidase 9 X-linked (*USP9X*) (8%), with the c.181G > T (p.E61*), c.3312dupA (p.P1105Tfs*4), c.4603 + 2 T > C (p.X1535_splice), c.5393A > C (p.K1798T) mutations.

Other less frequently mutated PTC-associated genes were reported in Additional file 5: Table S5.

We also reported altered pathways in different PTC tumor stages. The RTK/RAS/MAP kinase signaling pathway (hereafter RTK/RAS) was the most frequently affected by somatic mutations in all stages. The overall results were reported in supplementary Additional file 6: Figure S1.

### Mutational processes vary dynamically during tumor evolution

**Fig. 4:**
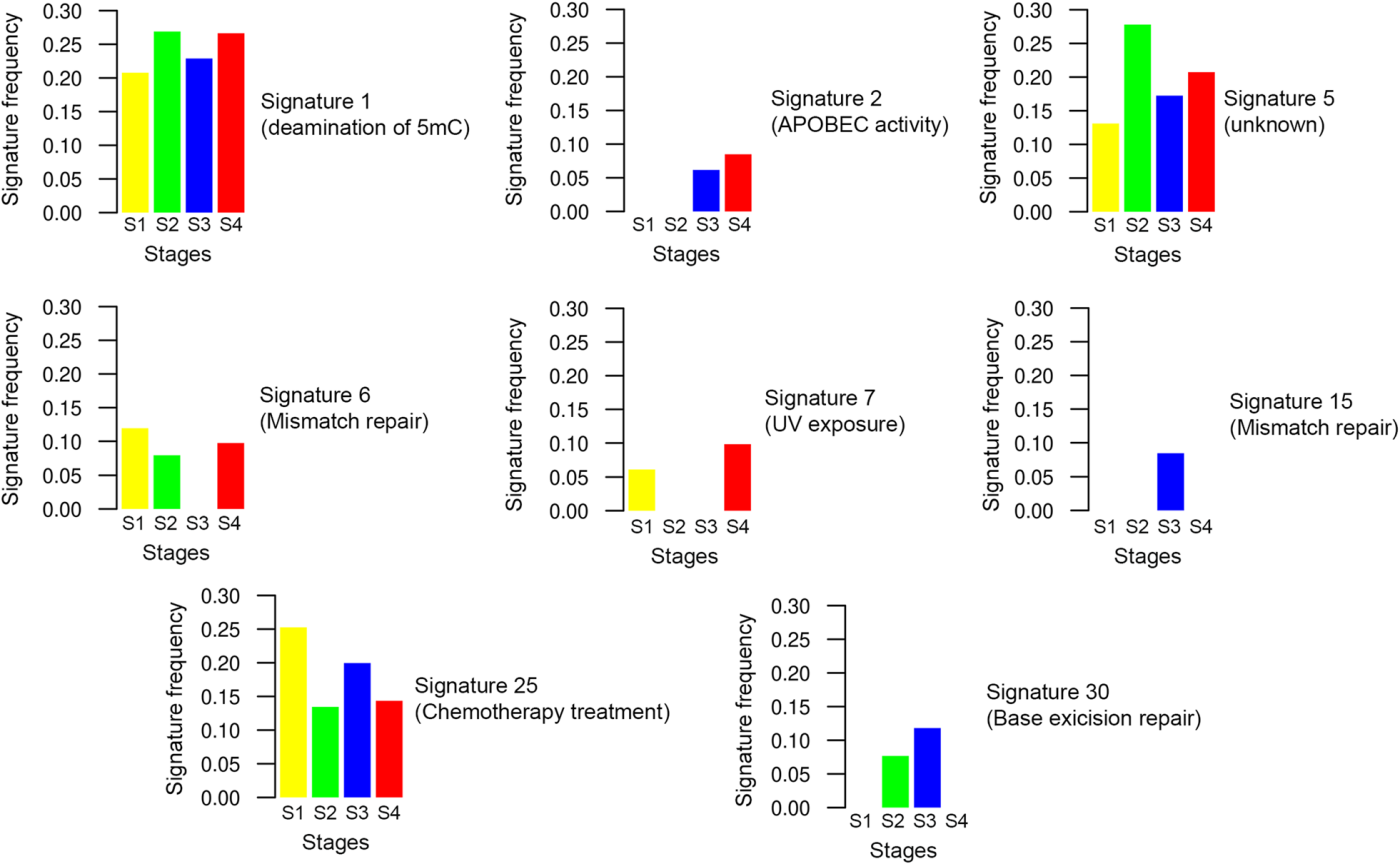
Mutational signatures in different stages of PTC. Barplot shows the weighted contributions of mutation signatures in the 4 tumor stages. Bars colors indicate the 4 tumor stages. Vertical axes depict the mutational signature frequency in each tumor stage. Signatures indicate as “unknown” has an undetermined etiology (see “Results” section for more details)

Somatic alterations in cancer genome may provide insights into the mutational processes occurring during tumorigenesis. These processes leave a peculiar pattern of mutations, called as mutational signatures [32]. We analyzed these mutational signatures across tumor stages to determine the mutational processes contributing to intratumor heterogeneity and shaping PTC tumor evolution. We identified two mutational signatures shared among all tumor stages (Fig. 4): Signature 1, related to endogenous spontaneous deamination of 5-methylcytosines, and Signature 25, related to chemotherapy treatment. While the Signature 25 was prominent in stage 1 and decreased in stage 4, the Signature 1 had an opposite trend. Furthermore, the Signature 5 was enriched in stage 2, although the etiologies of this signature have not been elucidated. Although its etiology is unknown, Signature 5 shows a clock-like behavior in many cancer types in that the number of mutations increases with the age [42]. Moreover, Signature 5 exhibits transcriptional strand bias for T > C substitutions, potentially indicating that some of these mutations arise from adducts subject to transcription-coupled repair [43]. In addition, we found that distinct defective DNA repair mechanisms might play a role in the tumor progression towards more aggressive stages. Indeed, we found a contribution of DNA mismatch repair (MMR) deficiency signatures (6, 15) in all tumor stages. Signatures 6 and 15 are two of the seven mutational signatures associated with defective MMR and microsatellite instability (MSI). As seen in more aggressive forms of thyroid cancer [44], tumors harboring MMR deficiency signatures completely lack loss-of-function mutations in MMR genes (*MLH1*, *MSH2* and *MSH6*). In addition, mutations in stages 2 and 3 were associated with Signature 30, related to defective DNA base excision repair due to inactivating mutations in *NTHL1*, while mutations in stages 1 and 4 were associated with Signature 7, related to Ultraviolet light exposure. Finally, advanced tumor stages (stage 3 and stage 4) were characterized by mutations assigned to Signature 2, associated with the activity of APOBEC family of cytidine deaminase.

### Common mutated genes in 4 tumor stages

To characterize changes in the genetic architecture of PTC, we extracted the 12 common mutated genes across clinical stages, regardless of mutation type. We identified three patterns depending on the behaviors of these genes’ mutation changes: (i) recurrently mutated genes in all the stages; (ii) some genes mostly mutated in early stages while disappeared in later stages; (iii) other mutated genes emerged in a dominant way in advanced stages.

**Fig. 5:**
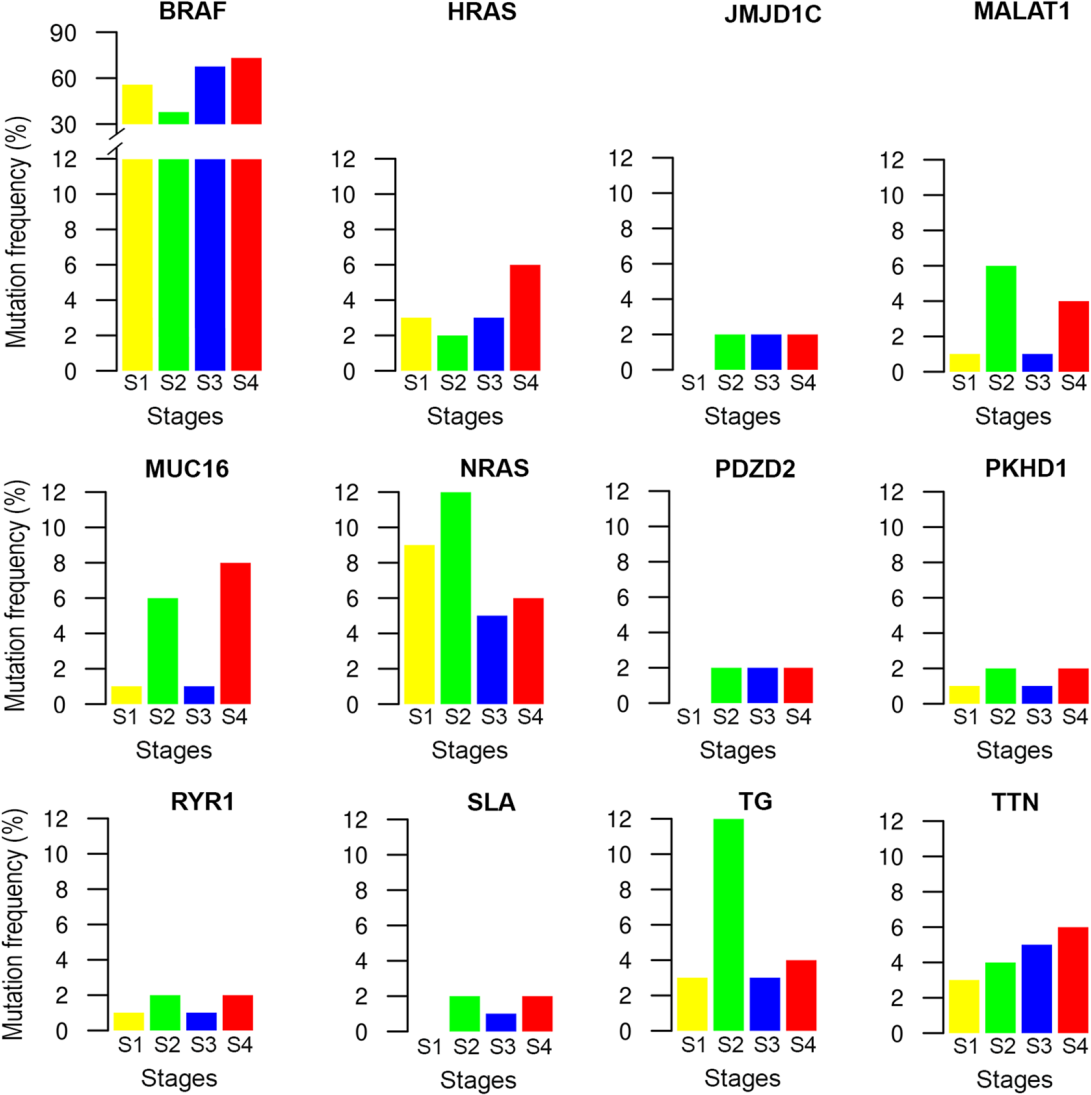
Mutation Frequency of common genes across clinical stages. For each gene in each tumor stage—indicated with bars of different colors—the mutation frequency is shown. Note that the maximum range is 12% for all genes and, in addition, the barplot for BRAF has a secondary y-axis to show its maximum mutation frequency in different tumor stages

In the first scenario, we found the *BRAF* gene, with the lowest mutation frequency in stage 2 (38%) and the highest one in stage 4 (75%). However, compared to other mutated genes, it has the highest mutation rate in all clinical stages (Fig. 5).

In the second scenario, we found genes mostly mutated in the early stages (stage 1 and stage 2). Specifically, in stage 1, the dominant mutated gene was *NRAS* (9%); its frequency decreased in stage 3 (5%) and stage 4 (6%). *JMJD1C* and *PDZD2* reached a plateau from stage 2 with a mutation frequency of 2% and remained constant in advanced stages (Fig. 5). In stage 2, the dominant mutated genes were *NRAS* (12%) and *TG* (12%) genes. Both of them decreased in advanced stages: *NRAS* had a mutation frequency of 5% in stage 3 and 6% in stage 4, while *TG* had a mutation frequency of 3% in stage 3 and 4% in stage 4 (Fig. 5).

In the last scenario, we found genes mostly mutated in advanced stages (stage 3 and stage 4). In stage 3, the dominant mutated genes were *NRAS* (5%) and *TTN* (5%). Both of them had a tendency to slightly increase in stage 4 (6% for *NRAS* and 6% for *TTN*) (Fig. 5). In stage 4, the dominant mutated genes were *MUC16* (8%), followed by *HRAS* and *TTN* genes showing a similar mutation frequency (about 6%).

Taken together, our results suggest that: (i) *BRAF* driver gene shows a dominant or “wave” role across all stages, (ii) one to three driver genes exhibit a dominant role in certain stages; (iii) common genes, differently mutated across clinical stages, drive the cancer cells developing from early to advanced stages and highlight the dynamic changes of PTC oncogenesis (Fig. 5).

It is noted that mutation frequency changes were also sometimes associated with a different codon change (Table 5). Indeed, the codon changes also showed certain dynamics across stages. Specifically, some codon changes highly occurred in all stages in specific hotspots positions, such as the c.1799 T > A (p.V600E) mutation in *BRAF* gene, the c.182A > G (p.Q61R) mutation in *HRAS* gene, and the c.182A > G (p.Q61R) and c.181C > A (p.Q61K) mutations in *NRAS* gene. For the other genes, different codon changes dominated in different stages.

**Table 5:** Mutation rate and codon changes of the common mutated genes in each tumor stage

Gene	Stage 1		Stage 2		Stage 3		Stage 4	
	Mut.Rate (%)	HGVSc (HGVSp)	Mut.Rate (%)	HGVSc (HGVSp)	Mut.Rate (%)	HGVSc (HGVSp)	Mut.Rate (%)	HGVSc (HGVSp)
BRAF	57	c.1799 T > A (p.V600E)	38	c.1799 T > A (p.V600E)	69	c.1799 T > A (p.V600E)	75	c.1799 T > A (p.V600E)
		c.1801A > G (p.K601E)		c.1801A > G (p.K601E)				
		c.1800G > A (p.V600V)						
		c.1467_1481delACCTACACCTCAGCA (p.P490_Q494del)						
HRAS	3	c.182A > G (p.Q61R)	2	c.182A > G (p.Q61R)	3	c.182A > G (p.Q61R)	6	c.182A > G (p.Q61R)
		c.181C > A (p.Q61K)				c.181C > A (p.Q61K)		c.181C > A (p.Q61K)
JMJD1C	0	c.4402C > T (p.Q1468*)	2		2		2	
				c.2203C > T (p.Q735*)				
						c.2014A > T (p.R672*)		
						c.3491delC (p.P1164Qfs*13)		
								c.2241C > A (p.T747T)
MALAT1	1		6		1		4	
MUC16	1	c.17957C > A (p.A5986E)	6		1		8	
		c.32628A > T (p.G10876G)						
		c.41805G > A (p.L13935L)						
				c.12963 T > C (p.A4321A)				
				c.23070G > A (p.P7690P)				
				c.7382C > A (p.T2461K)				
						c.12466A > G (p.T4156A)		
								c.1295C > A (p.T432N)
								c.18018C > A (p.H6006Q)
								c.22026A > C (p.T7342T)
								c.32174C > G (p.P10725R)
NRAS	9	c.182A > G (p.Q61R)	12	c.182A > G (p.Q61R)	5	c.182A > G (p.Q61R)	6	c.182A > G (p.Q61R)
		c.181C > A (p.Q61K)		c.181C > A (p.Q61K)		c.181C > A (p.Q61K)		c.181C > A (p.Q61K)
PDZD2	0	c.2039C > G (p.P680R)	2		2		2	
				c.805G > A (p.G269S)				
						c.3424_3427delACAG (p.T1142*)		
						c.3887C > A (p.A1296E)		
								c.937G > T (p.G313C)
PKHD1	1	c.3197C > T (p.S1066L)	2		1		2	
		c.8549C > A (p.T2850K)						
				c.8671C > T (p.R2891C)				
						c.1674C > T (p.L558L)		
								c.3568C > T (p.L1190F)
RYR1	1	c.3206A > T (p.D1069V)	2		1		2	
		c.3575G > T (p.S1192I)						
				c.9858G > C (p.E3286D)				
						c.6244G > A (p.E2082K)		
								c.5335C > G (p.P1779A)
SLA	0	c.225A > G (p.I75M)	2		1		2	
				c.-3_1delGAAA (NA)				
						c.694A > C (p.S232R)		
						c.695G > A (p.S232N)		
						c.696C > T (p.S232S)		
								c.645_646delGA (p.N216Pfs*26)
TG	3	c.2037C > T (p.G679G)	12		3		4	
		c.2462_2466delTTCAA (p.I821Kfs*4)						
		c.3737A > C (p.Q1246P)						
		c.3917G > C (p.C1306S)						
		c.4847_4853dupTCACCGT (p.S1619Hfs*12)						
		c.5673dupA (p.W1892Mfs*38)						
		c.5928_5929dupAT (p.S1977Yfs*37)						
		c.6844_6847delTTGT (p.L2282Ifs*61)						
				c.1038dupG (p.H347Afs*14)				
				c.1663G > A (p.E555K)				
				c.450G > T (p.G150G)				
				c.490 T > G (p.C164G)				
				c.5707_5708dupTT (p.C1904Sfs*7)				
				c.666 T > C (p.S222S)				
						c.1531_1534delAATG (p.N511Efs*15)		
						c.2412G > A (p.V804V)		
						c.84C > T (p.A28A)		
								c.418 T > G (p.C140G)
								c.4544_4546delAGA (p.Q1515del)
TTN	3	c.10361-3907 T > C (NA)	4		5		6	
		c.10361-4179G > A (NA)						
		c.34264 + 5360G > T (NA)						
		c.34264 + 578A > G (NA)						
		c.56257A > C (p.K18753Q)						
		c.57311_57314delCAAA (p.T19104Rfs*10)						
		c.62135-36delA (NA)						
				c.7061G > A (p.R2354H)				
				c.97072 T > A (p.W32358R)				
						c.24810G > C (p.E8270D)		
						c.68948C > T (p.T22983I)		
						c.83902C > T (p.R27968*)		
						c.97262 T > C (p.M32421T)		
						c.9769C > T (p.R3257C)		
								c.10360 + 5384 T > C (NA)
								c.45373C > T (p.R15125*)
								c.91311C > T (p.Y30437Y)

### Expression level analysis using web resource

**Fig. 6:**
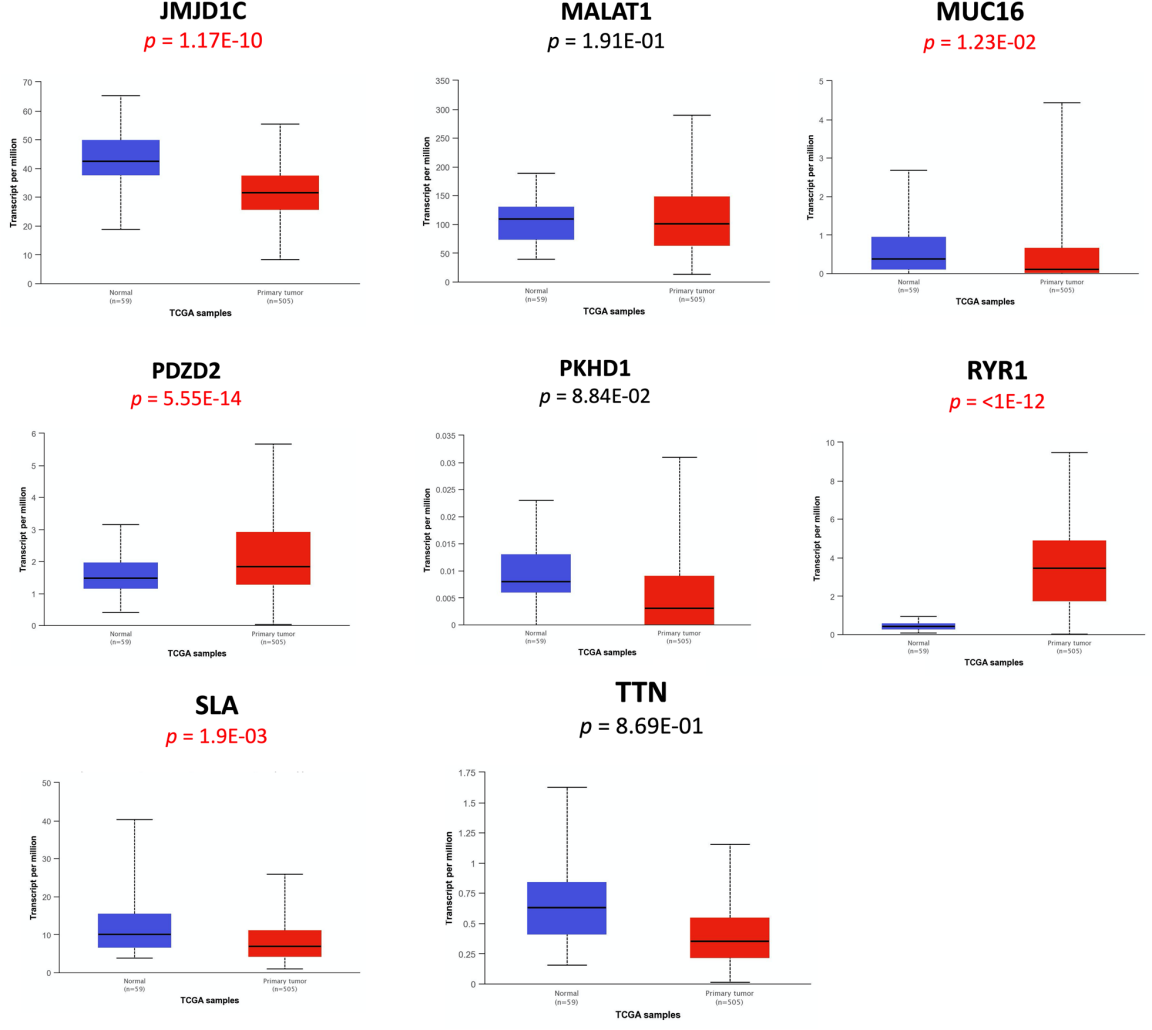
Expression levels of the common mutated genes in human thyroid cancers. Gene expression level of *JMJD1C*, *MALAT1*, *MUC16*, *PDZD2*, *PKHD1*, *RYR1*, *SLA* and *TTN* was analyzed in the TCGA database using UALCAN data portal. Box-Whisker plots show the expression level of the indicated genes in 59 normal thyroid tissues (in blue) and in 505 primary thyroid tumors (in red). Significant differences, estimated by Student’s t-test, have been highlighted in red characters

We aimed to evaluate the common mutated genes (*JMJD1C*, *MALAT1*, *MUC16*, *PDZD2*, *PKHD1*, *RYR1*, *SLA* and *TTN*) between thyroid tumor and normal samples. To this aim, we performed an in-silico analysis with UALCAN interactive computational tool [40]. The UALCAN data portal generated boxplots for each gene across 505 primary tumors and 59 normal thyroid tissues. The analysis showed that the gene expression level of *JMJD1C*, *MUC16* and *SLA* were statistically down-regulated while the gene expression level of *PDZD2* and *RYR1* were up-regulated in thyroid cancer with respect to normal thyroid tissues (Fig. 6).

**Fig. 7:**
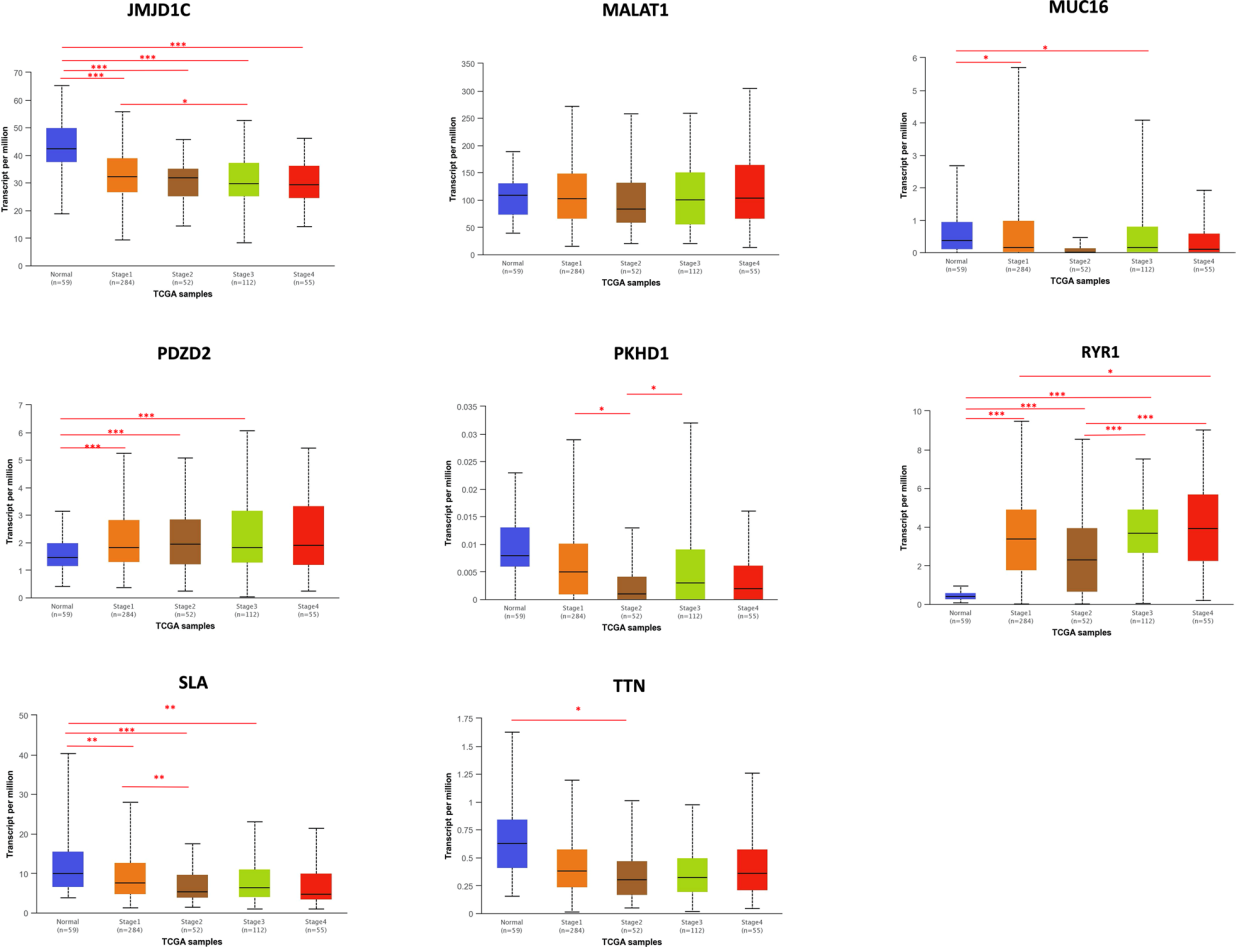
Expression levels of the common mutated genes based on thyroid cancer stages. Gene expression of *JMJD1C*, *MALAT1*, *MUC16*, *PDZD2*, *PKHD1*, *RYR1*, *SLA* and *TTN* was analyzed across the different thyroid cancer stages deposited in the TCGA project using UALCAN data portal. Box-Whisker plots show the expression level of the indicated genes in 59 normal thyroid tissues (in blue), 284 PTC in stage 1 (in orange), 52 PTC in stage 2 (in brown), 112 PTC in stage 3 (in green) and 55 PTC in stage 4 (in red). Statistical differences were calculated using Student’s t-test. *p ≤ 0.05; **p ≤ 0.01; ***p ≤ 0.001

We also compared the expression of these genes across different cancer stages. Based on AJCC (American Joint Committee on Cancer) 284 patients were in stage 1, 52 in stage 2, 112 in stage 3 and 55 in stage 4. Box-whisker plots in Fig. 7 showed that *JMJD1C*, *PDZD2*, *PKHD1*, *RYR1* and *SLA* presented a different expression among thyroid cancer stages, suggesting that the expression of these genes could reflect the intra-tumoral genetic heterogeneity across specific stages. Interestingly, since *RYR1* was differentially expressed across all the stages, it is possible that this gene could play a key role in the pathogenesis of thyroid cancer.

### Expression level of the common mutated genes in human thyroid cancer cell lines

**Fig. 8:**
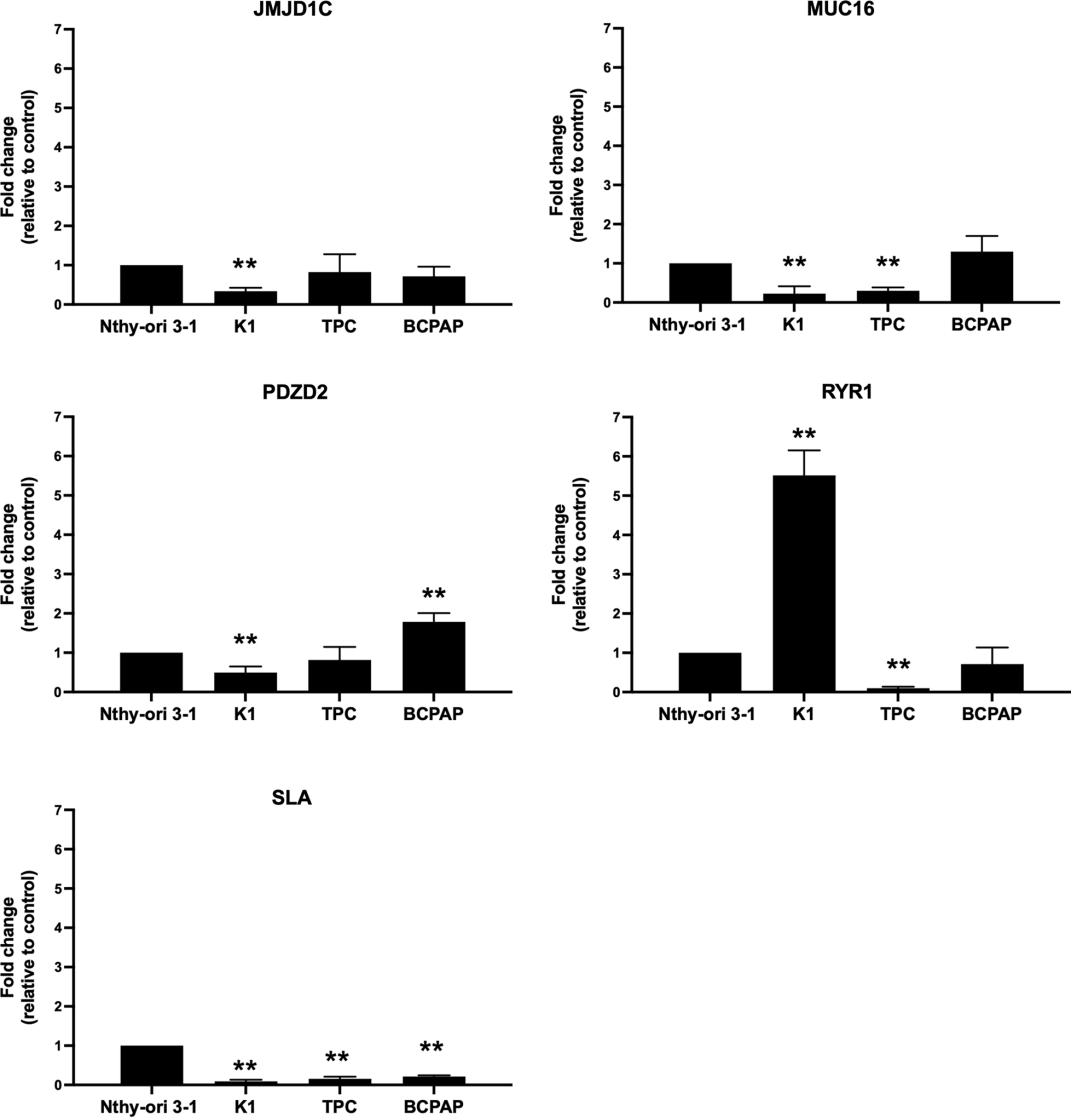
Expression levels of the common mutated genes in human thyroid cancer cell lines. Gene expression of *JMJD1C*, *MUC16*, *PDZD2*, *RYR1* and *SLA* was analyzed in immortalized normal thyroid epithelial (Nthy-ori 3-1) and papillary thyroid cancer cell lines (K1, TPC-1 and BCPAP) by qRT-PCR. Fold‐change was determined by 2^−ΔΔCt^ formula and β‐actin was used as internal control. Results are the means of three independent experiments ± standard error of the mean (SEM). **p ≤ 0.01

Finally, we investigated the expression levels of the common mutated genes (*JMJD1C*, *MUC16*, *PDZD2*, *RYR1*, and *SLA*) in papillary thyroid cancer cell lines (K1, TPC-1 and BCPAP) compared to an immortalized normal thyroid epithelial cell line (Nthy-ori 3-1) by qRT-PCR. We found a different expression levels of these genes among the different cell lines (Fig. 8). In line with our previous findings based on TCGA, *RYR1* was highly expressed in an aggressive cell line, K1, derived from metastasis of a well-differentiated PTC compared to Nthy-ori 3-1 (Fig. 8).

Finally, to verify whether the different expression level of the investigated genes in the thyroid cancer cell lines was due to their different mutational status, we purified the genomic DNA from cultured cells and sequenced the exonic region of the genes carrying high impact mutations (Table 5). We found that the high impact mutations in *JMJD1C*, *SLA*, *PDZD2*, identified from in-silico analysis in PTC samples, are missing in the thyroid cancer cell lines analyzed, thus suggesting that the altered expression of these genes is due to other regulative biological mechanisms.

## Discussion

Tumor heterogeneity results from the continuous accumulation of mutations during disease progression, leading to the occurrence of genetically different tumor subpopulations and influencing the clinical outcome of patients [1, 4, 5, 45]. Due to tumor heterogeneity, the mutational landscape may differ considerably among different clinical stages of the same tumor. Based on literature results, our study is the first to directly address the evolution of intra-tumoral genetic heterogeneity during PTC progression across tumor stages. Using the whole-exome sequencing data from the TCGA-THCA cohort, we tracked the intra-tumoral heterogeneity and assessed its clinical relevance through different stages of the PTC progression to better understand the genetics of PTC carcinogenesis. We also revealed the timing of mutational processes and the dynamics of the temporal acquisition of somatic events during the lifetime of the PTC. Finally, we assayed the relationship between the mutational status and RNA expression level in a panel of thyroid cancer cell lines. Overall, the findings support the occurrence of the genetic instability in tumor progression so that the mutational landscape of each tumor stage is not stable; rather, it is subject to periodic fluctuations and multiple mutational processes shape the mutational spectra of the lesion. This appears to occur in a temporal-specific manner, thus painting a stage-specific mutational picture in a continuous dynamic manner and determining ITH to some extent. The expression levels of selected genes also showed a different expression level among cell lines characterized by different origins (primary PTC or metastasis from PTC) and by different mutation status [46].

Pan-cancer studies have shown that ITH impacts the clinical outcome and contributes to the risk of poor survival in tumor patients [14, 15]. However, the association between ITH, measured by MATH score, and different tumor stages of PTC have not been thoroughly explored. Our study represents the first attempt to describe the prognostic value of MATH score. We found that ITH significantly influences the PTC patient’s survival rate and as genetic heterogeneity increases, the prognosis gets worse in advanced tumor stages, mainly in stage 4. A high MATH score indicates a high percentage of heterogeneous subclones [47], which also likely makes the tumor more aggressive. Indeed, high MATH scores were associated with advanced-stage tumors and shorter overall survival in head and neck squamous cell carcinoma [10, 11], with tumor stage and triple-negative or basal-like subtypes in breast cancer [48, 49] and with higher risks of metastasis in stage 2 and 3 colon cancer [50]. Moreover, a high MATH score was a potential unfavorable prognostic factor in PTC, not affected by any other clinic-pathologic features. Conversely, the MATH value was not the only factor affecting the prognosis in patients with low tumor heterogeneity. Indeed, the low MATH score showed a potential M stage-dependent prognostic power, thus associated with a worse prognosis when the tumor in an advanced stage spreads to distant organs and tissues. Although the mechanisms underlying the links between high genetic heterogeneity and short overall survival cannot be deduced from these results, the strong association between ITH and disease stage supports a possible role for ITH as a prognostic biomarker in PTC. Further investigation will be required to use ITH as a novel potential biomarker for survival prediction and therapy selection.

Both somatic mutations and mutational processes can account for this heterogeneity, providing mutational fuel upon which selection can act. Some of these mutational processes are active throughout the lifetime of the cancer cell while other ones are active in a temporal-specific manner [42, 51]. In accordance with this evidence, we found that the PTC genome harbored a mixture of signatures from different mutational processes. Multiple endogenous or exogenous mutational forces can operate simultaneously or successively in tumor stage diversification during PTC progression. Worthy of note is that several mutational signatures related to DNA repair deficiency (APOBEC-mediated mutagenesis, mismatch repair and base excision repair) increased later in tumor evolution or were specific for advanced stages [52], thus supporting the notion that biological processes driving the development of PTC may differ from those resulting in their progression.

The well-known driver genes (*BRAF*, *RAS*) belonging to MAPK or PI3K pathways [18–21] harbored the naturally occurring mutations and were commonly mutated across tumor stages. Specifically, mutations in *BRAF* and *RAS* were established at the early stages and confirmed in advanced stages; thus they can be considered early clonal events. On the other hand, some genes with moderate frequency emerged with a stage-by-stage expansion. Therefore, a rapidly growing clone may arise from several early key mutations, with subsequent mutations improving the fitness of tumor cell subpopulations in each stage. These subclonal alterations may likely predispose to the advancement towards more aggressive stages and potentially poorer prognosis tumors.

To better characterize the changing genetic architecture of PTC and highlight the dynamic changes of PTC oncogenesis, we followed the mutational patterns of 12 common mutated genes across clinical stages, regardless of mutation type. We identified three evolutionary paths of gene mutations that could drive PTC progression across pathological stages: (i) recurrently mutated genes in all the stages, with *BRAF* driver gene showing a dominant role across all stages; (ii) some genes (*NRAS* and *TG*) mostly mutated in the early stages while disappearing in the advanced stages; (iii) other mutated genes (*TTN*, *MUC16* and *HRAS*) emerged dominantly in advanced stages, thus suggesting their impact in the aggressive phenotype of this tumor. It also appeared that distinct somatic events affecting the same gene occurred in distinct tumor subclones. Indeed, the mutation frequency changes, sometimes associated with a different codon change, also showed certain dynamics across stages. Specifically, some codon changes highly occurred in all stages in specific hotspots positions (*BRAF* c.1799 T > A, *HRAS* c.182A > G, *NRAS* c.182A > G and c.181C > A) while for the other genes, different codon changes dominated in various stages. The theory of clonal evolution of tumor [5] argues that the accumulation of mutations drives early slow-growing subclones into fast-growing subclones, thus accelerating the tumor progression. In accordance with this theory, mutations occurring at hotspot positions may drive the PTC progression towards more aggressive stages. Meanwhile, each stage acquires a specific mutational landscape that does not directly drive cancer progression but may have a strong cumulative effect, thus contributing to the aggressiveness of cancer.

Except for *BRAF*, *HRAS*, *NRAS* and *TG*, whose involvement in PTC is deeply revealed [18–21], for the other commonly mutated genes in all tumor stages, we further assayed the relationship between TCGA data, the RNA expression level of thyroid cancer-derived cell lines and their mutational status. We found different expression levels of the common mutated genes among cell lines. These differences were not due to high impact mutations harboring by these genes but probably to additional altered mechanisms such as transcriptional regulatory networks (e.g., expression of miRNAs and transcriptional factors) and epigenetic networks (e.g., DNA methylation or chromatin remodeling complex). Additionally, the BCPAP cell line is derived from a poorly differentiated PTC; the TPC-1 cell line is derived from differentiated PTC, and K1 cell line derives from metastasis of a well-differentiated PTC. Also, the mutation status of these cell lines is different: BCPAP harbored *BRAF* and *TP53* mutations, TPC-1 cells harbored the *RET/PTC1* gene rearrangement, and K1 harbored mutations in *PIK3CA* and *BRAF* genes [46]. All these data confirmed the intra-tumoral genetic heterogeneity across different papillary thyroid carcinoma models. In accordance with our results, Shen et al. [53] found an up-regulation of the *RYR1* gene in 100 thyroid cancer samples compared to 64 normal thyroid tissue samples. RYR1 protein belongs to ryanodine receptors family and acts as a calcium (Ca^2+^) release channel. Since the altered homeostasis of intracellular Ca^2+^ is correlated to several hallmarks of cancer cells, the study of *RYR1* could be interesting to better understand the pathogenesis of thyroid cancer. Although it is not clear how these mutations regulate RNA expression levels, the here screened genes were meaningful and worthy of further studies to better investigate the functional mechanism of how mutations work.

One of the most notable strengths of this study is the stratification of patients by tumor stage. This allowed us to obtain homogeneous patient subpopulations to be profiled for mutational landscape. However, our study has several limitations. First, we used samples taken from one small area of the tumor and at one point in the disease course. The analysis of a single biopsy could not be representative of the whole tumor. Thus, we likely underestimated the true extent of heterogeneity within each tumor stage. Comprehensive longitudinal studies, coupled with deep multi-region sequencing, are required to better understand the mutational evolution of PTC over time. Second, our study is a retrospective analysis of a single publicly available data. Thus, a multi-data analysis and a prospective study will be needed to validate the prognostic value of the MATH score and the mutational processes driving the PTC progression towards more advanced and aggressive tumor stages. Thirdly, further in-depth studies in vitro and/or in vivo are required to gain a deeper understanding of the functional impact of *JMJD1C*, *MALAT1*, *MUC16*, *PDZD2*, *PKHD1*, *RYR1*, *SLA* and *TTN* on changes of RNA expression levels. As such, our results should be considered as hypothesis-generating findings.

Despite these limitations, our results improve the knowledge of intra-tumoral heterogeneity in different tumor stages of PTC, revealing for the first time that ITH measured by MATH could be a potential unfavorable prognostic factor in PTC and a potential risk factor for shorter survival. Moreover, this study shed light that different biological processes contributed to tumor heterogeneity of PTC, by both adding to the mutational burden and promoting molecular diversification of PTC in different tumor stages. In conclusion, our study may contribute to the development of precision medicine and the improvement of diagnostic strategies in PTC patients.

## Conclusions

Emerging data, mainly due to new technologies, showed that the intra-tumoral heterogeneity represents an important feature of human cancer. Our research unveiled that the intra-tumoral heterogeneity characterized the PTC aggressiveness among different stages opening new scenarios about its impact on diagnosis, prognosis and treatment of PTC patients. Indeed, the high ITH profile associated with advanced clinical stages could be responsible for the drug resistance, thus posing clinical challenges.

The identification of specific genetic alterations in PTC patients could represent a novel tool capable to increase the diagnostic sensitivity, providing novel implications for therapeutic decisions. Thus, the implication of this work could be important in clinical practice and highlight that pathologists should consider the ITH for a precise diagnosis. The findings of this work could also give insight into the molecular mechanisms associated with the progression from the less to the more aggressive form of PTCs.

## Supplementary Information

**Additional file 1: Table S1.** Genomic DNA sequencing of high impact mutations in common mutated genes in thyroid cancer cell lines. The genes, the exons harboring the high impact mutations found in PTC patients, and the primer sequences used are listed.

**Additional file 2: Table S2.** Clinical and pathological characteristics of patients in TCGA papillary thyroid carcinoma cohort. For each clinical feature, patients labeled as “Not Available” or “Unknown” are not shown.

**Additional file 3: Table S3.** Relationship of clinical variables with progression free survival (PFS) by univariate Cox proportional hazards analysis in low-MATH group. Statistical significance of differences between Kaplan–Meier survival curves was assessed by Log-rank test. Statistical relevance as prognostic value was assessed by Wald test.

**Additional file 4: Table S4.** Relationship of clinical variables with progression free survival (PFS) by univariate Cox proportional hazards analysis in high-MATH group. Statistical significance of differences between Kaplan–Meier survival curves was assessed by Log-rank test. Statistical relevance as prognostic value was assessed by Wald test.

**Additional file 5: Table S5.** Top 20 mutated genes in each tumor stage of PTC with the corresponding annotation.

**Additional file 6: Figure S1.** Altered pathways in different tumor stages in PTC.

## Data Availability

Clinical information and mutation data of THCA are available from the public databases cBioPortal (http://www.cbioportal.org/) and TCGA (https://portal.gdc.cancer.gov/), respectively.

## Abbreviations

BRAF: B-Raf proto-oncogene, serine/threonine kinase
HRAS: HRas proto-oncogene, GTPase
ITH: Intra-tumor heterogeneity
JMJD1C: Jumonji domain containing 1C
KRAS: KRAS proto-oncogene, GTPase
MALAT1: Metastasis associated lung adenocarcinoma transcript 1
MAPK: Mitogen-activated protein kinase
MATH: Mutant allele tumor heterogeneity
MUC16: Mucin 16
NRAS: NRAS proto-oncogene, GTPase
PDZD2: PDZ domain containing 2
PFS: Progression-free survival
PKHD1: PKHD1 ciliary IPT domain containing fibrocystin/polyductin
PTC: Papillary thyroid carcinoma
RTK: Receptor tyrosine kinase
RYR1: Ryanodine receptor 1
SLA: Src like adaptor
SNV: Single nucleotide variant
TCGA: The Cancer Genome Atlas
TG: Thyroglobulin
THCA: Thyroid cancer
TTN: Titin
