# Supplementary material for: Evolution of intra-tumoral heterogeneity across different pathological stages in papillary thyroid carcinoma
Source: Cancer Cell Int. 2022 Aug 22;22:263. doi: 10.1186/s12935-022-02680-1 (PMC9394008; doi:10.1186/s12935-022-02680-1)
Supplement: Supplementary file 1 — Additional file 1: Table S1. Genomic DNA sequencing of high impact mutations in common mutated genes in thyroid cancer cell lines. The genes, the exons harboring the high impact mutations found in PTC patients, and the primer sequences used are listed. [file 12935_2022_2680_MOESM1_ESM.docx]

**Table S1. Genomic DNA sequencing of high impact mutations in common mutated genes in thyroid cancer cell lines.** The genes, the exons harboring the high impact mutations found in PTC patients, and the primer sequences used are listed.

| Gene | Exon | Mutation | Forward (5’-3’) | Reverse (5’-3’) |
| --- | --- | --- | --- | --- |
| JMJD1C | 10 | c.3491delC | GTGCCTCAGAGTTTACCCCA | AGGCTGTGATGGATTGGTGG |
|  |  | c.4402C>T | GCTGCCGATACTACCAGTGT | GTTGTGCCTGAGAACCCAGA |
|  | 8 | c.2203C>T | CCAGCCCATCACCTGAAGTT | GTTGTTGTTGCTGGTGTGCT |
|  |  | c.2014A>T |  |  |
| SLA | 9 | c.645_646delGA | TCTCTACTGCCTTTTCTTCTGGC | TCTCTTGCTGCCACCATACA |
| PDZD2 | 18 | c.3424_3427delACAG | AGTCCGTACAGACACCCAGA | AGCCATCATCCTTTCCCACTG |
