# Supplementary material for: Evolution of intra-tumoral heterogeneity across different pathological stages in papillary thyroid carcinoma
Source: Cancer Cell Int. 2022 Aug 22;22:263. doi: 10.1186/s12935-022-02680-1 (PMC9394008; doi:10.1186/s12935-022-02680-1)
Supplement: Supplementary file 2 — Additional file 2: Table S2. Clinical and pathological characteristics of patients in TCGA papillary thyroid carcinoma cohort. For each clinical feature, patients labeled as “Not Available” or “Unknown” are not shown. [file 12935_2022_2680_MOESM2_ESM.docx]

**Table S2. Clinical and pathological characteristics of patients in TCGA papillary thyroid carcinoma cohort.** For each clinical feature, patients labeled as “Not Available” or “Unknown” are not shown

| **Clinical Features** | **Category** | **PTC, *n*=474** |
| --- | --- | --- |
| **Thyroid Gland Disorder** ^(1)^ | Normal | 263 |
|  | Nodular Hyperplasia | 65 |
|  | Lymphocytic Thyroiditis | 42 |
| **Primary Neoplasm Anatomic Site** | Right Lobe | 203 |
|  | Left Lobe | 168 |
|  | Bilateral | 76 |
|  | Isthmus | 22 |
| **Histological Type** | Classical | 339 |
|  | Follicular (>= 99% follicular patterned) | 99 |
|  | Tall Cell (>= 50% tall cell features) | 36 |
| **Residual Tumor** | R0 | 369 |
|  | R1 | 48 |
|  | R2 | 4 |
|  | RX | 28 |
| **Extrathyroid Extension Status** | None | 316 |
|  | Minimal (T3) | 126 |
|  | Moderate/Advanced (T4a) | 17 |
|  | Very Advanced (T4b) | 1 |

^(1)^ Patients labeled as “Other, specify”, “Lymphocytic Thyroiditis|Nodular Hyperplasia”, “Lymphocytic Thyroiditis|Other, specify”, “Lymphocytic Thyroiditis|Nodular Hyperplasia|Other, specify “, “Normal|Other,specify” are not shown.
