# Supplementary material for: Evolution of intra-tumoral heterogeneity across different pathological stages in papillary thyroid carcinoma
Source: Cancer Cell Int. 2022 Aug 22;22:263. doi: 10.1186/s12935-022-02680-1 (PMC9394008; doi:10.1186/s12935-022-02680-1)
Supplement: Supplementary file 3 — Additional file 3: Table S3. Relationship of clinical variables with progression free survival (PFS) by univariate Cox proportional hazards analysis in low-MATH group. Statistical significance of differences between Kaplan–Meier survival curves was assessed by Log-rank test. Statistical relevance as prognostic value was assessed by Wald test. [file 12935_2022_2680_MOESM3_ESM.docx]

**Table S3. Relationship of clinical variables with progression free survival (PFS) by univariate Cox proportional hazards analysis in low-MATH group.** Statistical significance of differences between Kaplan-Meier survival curves was assessed by Log-rank test. Statistical relevance as prognostic value was assessed by Wald test.

|  | **Stage 1** | | | **Stage 2** | | | **Stage 3** | | | **Stage 4** | | | | |  |  |
| --- | --- | --- | --- | --- | --- | --- | --- | --- | --- | --- | --- | --- | --- | --- | --- | --- |
|  |  | **Univariate Cox analysis** | |  | **Univariate Cox analysis** | |  | **Univariate Cox analysis** | |  | | **Univariate Cox analysis** | | | |  |
| **Variables** | **P-value Log-rank test** | **HR**  **(95% CI)** | **P-value Wald Test** | **P-value Log-rank test** | **HR**  **(95% CI)** | **P-value Wald Test** | **P-value Log-rank test** | **HR**  **(95% CI)** | **P-value Wald Test** | **P-value Log-rank test** | | **HR**  **(95% CI)** | **P-value Wald Test** | | |  |
|  |  |  |  |  |  |  |  |  |  |  | |  |  | | |  |
| **Gender** | 1 |  |  | 0.5 |  |  | 0.6 |  |  | 0.2 | |  |  | | |  |
| Male vs Female |  | 1  (0.21 – 4.71) | 1 |  | 0  (0 - Inf) | 1 |  | 1.45  (0.36 - 5.88) | 0.61 |  | 3.41  (0.38 - 30.67) | | | 0.27 | | |
|  |  |  |  |  |  |  |  |  |  |  | |  |  | | |  |
| **Thyroid Gland Disorder** | 0.6 |  |  | 0.2 |  |  | 0.9 |  |  | 0.8 | |  |  | | |  |
| Lymphocytic Thyroiditis vs Normal |  | 1.74  (0.34 - 9.01) | 0.51 |  | - | - |  | 0  (0 - Inf) | 1 |  | | 0  (0 - Inf) | 1 | | |  |
| Nodular Hyperplasia vs Normal |  | 0  (0 - Inf) | 1 |  | 0  (0 - Inf) | 1 |  | 1.73  (0.18 - 16.71) | 0.64 |  | | 1.02  (0.11 - 9.29) | 0.98 | | |  |
|  |  |  |  |  |  |  |  |  |  |  | |  |  | | |  |
| **Primary Neoplasm Anatomic Site** | 0.7 |  |  | 0.07 |  |  | 0.2 |  |  | 0.06 | |  |  | | |  |
| Right Lobe vs Bilateral |  | 1.26  (0.15 - 10.77) | 0.84 |  | NA | NA |  | 1.55  (0.16 - 15.06) | 0.7 |  | | NA | NA | | |  |
| Left Lobe vs Bilateral |  | 0.72  (0.08 - 6.44) | 0.77 |  | 0.98  (0 - Inf) | 1 |  | 4.87  (0.55 - 43) | 0.15 |  | | NA | NA | | |  |
| Isthmus vs Bilateral |  | 0  (0 - Inf) | 1 |  | NA | NA |  | 0  (0 - Inf) | 1 |  | | -^(1)^ | -^(1)^ | | |  |
|  |  |  |  |  |  |  |  |  |  |  | |  |  | | |  |
| **Histological Type** | 0.2 |  |  | 0.8 |  |  | 0.6 |  |  | 0.6 | |  |  | | |  |
| Follicular vs Classical |  | 0.55  (0.07 - 4.41) | 0.57 |  | 0.77  (0.06 - 9.26) | 0.84 |  | 0.54  (0.06 - 4.6) | 0.57 |  | | 2.12  (0.24 - 19.01) | 0.5 | | |  |
| Tall Cell vs Classical |  | 4.33  (0.54 - 34.67) | 0.17 |  | - | - |  | 1.64  (0.39 - 6.96) | 0.5 |  | | 0  (0 - Inf) | 1 | | |  |
|  |  |  |  |  |  |  |  |  |  |  | |  |  | | |  |
| **T stage** | 0.8 |  |  | 0.6 |  |  | 0.5 |  |  | 0.7 | |  |  | | |  |
| T2 vs T1 |  | 1.41  (0.31 - 6.3) | 0.66 |  | NA | NA |  | 0  (0 - Inf) | 1 |  | | NA | NA | | |  |
| T3 vs T1 |  | 2.13  (0.43 - 10.62) | 0.36 |  | NA | NA |  | 1.38  (0.17 - 11.29) | 0.76 |  | | NA | NA | | |  |
| T4 vs T1 |  | 0  (0 - Inf) | 1 |  | - | - |  | 4.12  (0.25 - 67.23) | 0.32 |  | | NA | NA | | |  |
| TX vs T1 |  | - | - |  | - | - |  | - | - |  | |  |  | | |  |
|  |  |  |  |  |  |  |  |  |  |  | |  |  | | |  |
| **N stage** | 0.1 |  |  | 0.06 |  |  | 0.4 |  |  | 0.09 | |  |  | | |  |
| N1 vs N0 |  | 3.92  (0.79 - 19.45) | 0.09 |  | 14.87  (0.91 - 241.82) | 0.06 |  | 2.59  (0.54 - 12.52) | 0.24 |  | | 0.4  (0.06 - 2.63) | 0.34 | | |  |
| NX vs N0 |  | 5.57  (0.78 - 39.62) | 0.09 |  | 4.55  (0.28 - 73.24) | 0.29 |  | 0  (0 - Inf) | 1 |  | | 3.48  (0.25 - 47.69) | 0.35 | | |  |
|  |  |  |  |  |  |  |  |  |  |  | |  |  | | |  |
| **Residual Tumor** | 0.8 |  |  | 1 |  |  | 0.5 |  |  | 0.6 | |  |  | | |  |
| R1 vs R0 |  | 1.52  (0.19 - 12.02) | 0.69 |  | 0  (0 - Inf) | 1 |  | 2.53  (0.48 - 13.23) | 0.27 |  | | 0.84  (0.08 - 8.9) | 0.89 | | |  |
| R2 vs R0 |  |  |  |  | 0  (0 - Inf) | 1 |  | - | - |  | | 2.29  (0.36 - 14.44) | 0.38 | | |  |
| RX vs R0 |  | 0  (0 - Inf) | 1 |  | 0  (0 – GR/AH) | 1 |  | 2.09  (0.23 - 18.79) | 0.51 |  | |  |  | | |  |
|  |  |  |  |  |  |  |  |  |  |  | |  |  | | |  |
| **Extrathyroid Extension Status** | 0.9 |  |  | 0.8 |  |  | 0.4 |  |  | 1 | |  |  | | |  |
| Moderate/Advanced (T4a) vs Minimal (T3) |  | 0  (0 - Inf) | 1 |  | - | - |  | 2.86  (0.33 - 24.78) | 0.34 |  | | 0.74  (0.04 - 12.11) | 0.83 | | |  |
| Very Advanced (T4b) vs Minimal (T3) |  | - | - |  | - | - |  | - | - |  | | -^(1)^ | -^(1)^ | | |  |
| None vs Minimal (T3) |  | 0.85  (0.18 - 4.04) | 0.84 |  | 0.74  (0.06 - 8.64) | 0.81 |  | 0.54  (0.11 - 2.71) | 0.46 |  | | 0.93  (0.08 - 10.33) | 0.95 | | |  |
|  |  |  |  |  |  |  |  |  |  |  | |  |  | | |  |
| **Neoplasm width** | 1 |  |  | 0.4 |  |  | 0.4 |  |  | 0.06 | |  |  | | |  |
| Medium vs Low |  | 0.95  (0.16 - 5.67) | 0.95 |  | 0  (0 - Inf) | 1 |  | 0.98  (0.14 - 6.97) | 0.98 |  | | NA | NA | | |  |
| High vs Low |  | 1.16  (0.23 - 5.77) | 0.86 |  | 0  (0 - Inf) | 1 |  | 2.5  (0.46 - 13.64) | 0.29 |  | | NA | NA | | |  |
|  |  |  |  |  |  |  |  |  |  |  | |  |  | | |  |
| **Neoplasm depth** | 0.9 |  |  | 0.4 |  |  | 0.5 |  |  | 0.07 | |  |  | | |  |
| High vs Low |  | 1.08  (0.26 - 4.53) | 0.91 |  | 0  (0 - Inf) | 1 |  | 1.67  (0.4 - 6.99) | 0.48 |  | | 5.85  (0.68 - 50.21) | 0.11 | | |  |
|  |  |  |  |  |  |  |  |  |  |  | |  |  | | |  |

^(1)^ The Isthmus anatomic site and the very advanced (T4b) extrathyroid extension were removed from the analysis due to the occurrence of one single event.
