# Supplementary material for: Evolution of intra-tumoral heterogeneity across different pathological stages in papillary thyroid carcinoma
Source: Cancer Cell Int. 2022 Aug 22;22:263. doi: 10.1186/s12935-022-02680-1 (PMC9394008; doi:10.1186/s12935-022-02680-1)
Supplement: Supplementary file 4 — Additional file 4: Table S4. Relationship of clinical variables with progression free survival (PFS) by univariate Cox proportional hazards analysis in high-MATH group. Statistical significance of differences between Kaplan–Meier survival curves was assessed by Log-rank test. Statistical relevance as prognostic value was assessed by Wald test. [file 12935_2022_2680_MOESM4_ESM.docx]

**Table S4. Relationship of clinical variables with progression free survival (PFS) by univariate Cox proportional hazards analysis in high-MATH group.** Statistical significance of differences between Kaplan-Meier survival curves was assessed by Log-rank test. Statistical relevance as prognostic value was assessed by Wald test.

|  | **Stage 1** | | | **Stage 2** | | | **Stage 3** | | | **Stage 4** | | | | |  |  |
| --- | --- | --- | --- | --- | --- | --- | --- | --- | --- | --- | --- | --- | --- | --- | --- | --- |
|  |  | **Univariate Cox analysis** | |  | **Univariate Cox analysis** | |  | **Univariate Cox analysis** | |  | | **Univariate Cox analysis** | | | |  |
| **Variables** | **P-value Log-rank test** | **HR**  **(95% CI)** | **P-value Wald Test** | **P-value Log-rank test** | **HR**  **(95% CI)** | **P-value Wald Test** | **P-value Log-rank test** | **HR**  **(95% CI)** | **P-value Wald Test** | **P-value Log-rank test** | | **HR**  **(95% CI)** | **P-value Wald Test** | | |  |
|  |  |  |  |  |  |  |  |  |  |  | |  |  | | |  |
| **Gender** | 0.8 |  |  | 0.6 |  |  | 0.3 |  |  | 0.4 | |  |  | | |  |
| Male vs Female |  | 1.27  (0.25 – 6.32) | 0.77 |  | 2.16  (0.13 - 34.61) | 0.59 |  | 2.04  (0.48 - 8.62) | 0.33 |  | 0.53  (0.1 - 2.79) | | | 0.46 | | |
|  |  |  |  |  |  |  |  |  |  | 0.4 | |  |  | | |  |
| **Thyroid Gland Disorder** | 0.6 |  |  | 0.4 |  |  | 0.3 |  |  |  | |  |  | | |  |
| Lymphocytic Thyroiditis vs Normal |  | 0  (0 - Inf) | 1 |  | 0  (0 - Inf) | 1 |  | - | - |  | | - | - | | |  |
| Nodular Hyperplasia vs Normal |  | 0.86  (0.1 – 7.26) | 0.89 |  | 0  (0 - Inf) | 1 |  | 0  (0 - Inf) | 1 |  | | 2.5  (0.22 - 28.51) | 0.46 | | |  |
|  |  |  |  |  |  |  |  |  |  |  | |  |  | | |  |
| **Primary Neoplasm Anatomic Site** | 0.3 |  |  | 0.9 |  |  | 0.2 |  |  | 1 | | -^(1)^ | -^(1)^ | | |  |
| Right Lobe vs Bilateral |  | NA | NA |  | NA | NA |  | 0.24  (0.04 - 1.32) | 0.1 |  | | 1.2  (0.2 – 7.48) | 0.83 | | |  |
| Left Lobe vs Bilateral |  | NA | NA |  | 1.22  (0.08 - 19.86) | 0.89 |  | 0.29  (0.05 - 1.61) | 0.16 |  | | 1.02  (0.08 – 12.56) | 0.99 | | |  |
| Isthmus vs Bilateral |  | 1.03  (0 - Inf) | 1 |  | NA | NA |  | 0  (0 - Inf) | 1 |  | | - | - | | |  |
| **Histological Type** | 0.3 |  |  | 0.5 |  |  | 0.5 |  |  | 0.5 | |  |  | | |  |
| Follicular vs Classical |  | 1.89  (0.36 - 9.75) | 0.45 |  | 0  (0 - Inf) | 1 |  | 0  (0 - Inf) | 1 |  | | 1.86  (0.19 - 18.05) | 0.59 | | |  |
| Tall Cell vs Classical |  | 4.31  (0.49 - 38.21) | 0.19 |  | - | - |  | 0.61  (0.08 - 5) | 0.65 |  | | 3.95  (0.33 - 47.68) | 0.28 | | |  |
|  |  |  |  |  |  |  |  |  |  |  | |  |  | | |  |
| **T stage** | 0.7 |  |  | - |  |  | 0.9 |  |  | 0.2 | |  |  | | |  |
| T2 vs T1 |  | 1.93  (0.32 - 11.62) | 0.47 |  | - | - |  | NA | NA |  | | NA | NA | | |  |
| T3 vs T1 |  | 2.67  (0.44 - 15.98) | 0.28 |  | - | - |  | NA | NA |  | | NA | NA | | |  |
| T4 vs T1 |  | - | - |  | - | - |  | 1  (0 - Inf) | 1 |  | | NA | NA | | |  |
| TX vs T1 |  | 0  (0 - Inf) | 1 |  | - | - |  |  |  |  | | - | - | | |  |
|  |  |  |  |  |  |  |  |  |  |  | |  |  | | |  |
| **M stage** | 0.3 |  |  | 0.9 |  |  | 0.5 |  |  | 0.3 | |  |  | | |  |
| M1 vs M0 |  | - | - |  | - | - |  | - | - |  | | 5.19  (0.47 - 57.86) | 0.18 | | |  |
| MX vs M0 |  | 1.99  (0.47 - 8.47) | 0.35 |  | 1.22  (0.08 - 19.86) | 0.89 |  | 0.59  (0.12 - 2.92) | 0.52 |  | | 4.38  (0.41 - 46.88) | 0.22 | | |  |
|  |  |  |  |  |  |  |  |  |  |  | |  |  | | |  |
| **N stage** | 0.6 |  |  | 1 |  |  | 0.5 |  |  | 0.6 | |  |  | | |  |
| N1 vs N0 |  | 2.21  (0.4 - 12.05) | 0.36 |  | - | - |  | 0.62  (0.15 - 2.52) | 0.51 |  | | - | - | | |  |
| NX vs N0 |  | 2.37  (0.33 - 16.86) | 0.39 |  | NA | NA |  | 0  (0 - Inf) | 1 |  | | 0  (0 - Inf) | 1 | | |  |
|  |  |  |  |  |  |  |  |  |  |  | |  |  | | |  |
| **Residual Tumor** | 0.2 |  |  | 0.6 |  |  | 0.4 |  |  | 1 | |  |  | | |  |
| R1 vs R0 |  | 3.4  (0.68 - 16.87) | 0.13 |  | - | - |  | 2.39  (0.54 - 10.7) | 0.25 |  | | 0.92  (0.1 - 8.63) | 0.94 | | |  |
| R2 vs R0 |  |  |  |  | - | - |  | 4.33  (0.48 - 39.3) | 0.19 |  | | - | - | | |  |
| RX vs R0 |  | 0  (0 - Inf) | 1 |  | 0  (0 - Inf) | 1 |  | 0  (0 - Inf) | 1 |  | | 1.21  (0.13 - 11.5) | 0.87 | | |  |
|  |  |  |  |  |  |  |  |  |  |  | |  |  | | |  |
| **Extrathyroid Extension Status** | 0.6 |  |  | - |  |  | 0.8 |  |  | 0.3 | |  |  | | |  |
| Moderate/Advanced (T4a) vs Minimal (T3) |  | - | - |  | - | - |  | 0  (0 - Inf) | 1 |  | | 1.86  (0.17 - 20.75) | 0.62 | | |  |
| Very Advanced (T4b) vs Minimal (T3) |  | - | - |  | - | - |  | - | - |  | | - | - | | |  |
| None vs Minimal (T3) |  | 0.62  (0.13 - 3.09) | 0.56 |  | - | - |  | 0.69  (0.14 - 3.44) | 0.65 |  | | 5.54  (0.48 - 64.46) | 0.17 | | |  |
|  |  |  |  |  |  |  |  |  |  |  | |  |  | | |  |
| **Primary Neoplasm Focus Type** | 0.9 |  |  | 0.5 |  |  | 0.7 |  |  | 0.7 | |  |  | | |  |
| Unifocal vs Multifocal |  | 1.06  (0.25 - 4.56) | 0.93 |  | NA | NA |  | 0.72  (0.17 - 2.99) | 0.65 |  | | 1.33  (0.29 - 6.15) | 0.71 | | |  |
|  |  |  |  |  |  |  |  |  |  |  | |  |  | | |  |
| **Neoplasm Length** | 0.2 |  |  | 0.2 |  |  | 0.5 |  |  | 0.5 | |  |  | | |  |
| High vs Low |  | 2.73  (0.55 - 13.58) | 0.22 |  | NA | NA |  | 1.67  (0.37 - 7.49) | 0.5 |  | | 0.57  (0.12 - 2.6) | 0.46 | | |  |
|  |  |  |  |  |  |  | 0.6 |  |  |  | |  |  | | |  |
| **Neoplasm width** | 0.3 |  |  | 0.4 |  |  |  |  |  | 0.3 | |  |  | | |  |
| Medium vs Low |  | 4.74  (0.49 - 45.9) | 0.18 |  | 1  (0 - Inf) | 1 |  | 1.54  (0.26 - 9.27) | 0.64 |  | | 0.21  (0.02 - 1.89) | 0.16 | | |  |
| High vs Low |  | 3.54  (0.37 - 34.3) | 0.27 |  | NA | NA |  | 0.52  (0.05 - 5.78) | 0.59 |  | | 0.45  (0.07 - 2.76) | 0.39 | | |  |
|  |  |  |  |  |  |  |  |  |  |  | |  |  | | |  |
| **Neoplasm depth** | 0.2 |  |  | 0.5 |  |  |  |  |  | 0.4 | |  |  | | |  |
| High vs Low |  | 3.13  (0.61 - 16.14) | 0.17 |  | NA | NA | 0.4 | 0.52  (0.09 - 2.89) | 0.46 |  | | 0.45  (0.08 - 2.56) | 0.36 | | |  |
|  |  |  |  |  |  |  |  |  |  |  | |  |  | | |  |

^(1)^ The Isthmus anatomic site was removed from the analysis due to the occurrence of one single event.
