# Supplementary figures and images for: Evolution of intra-tumoral heterogeneity across different pathological stages in papillary thyroid carcinoma
Source: Cancer Cell Int. 2022 Aug 22;22:263. doi: 10.1186/s12935-022-02680-1 (PMC9394008; doi:10.1186/s12935-022-02680-1)

Figure S1. Altered pathways in different tumor stages in PTC

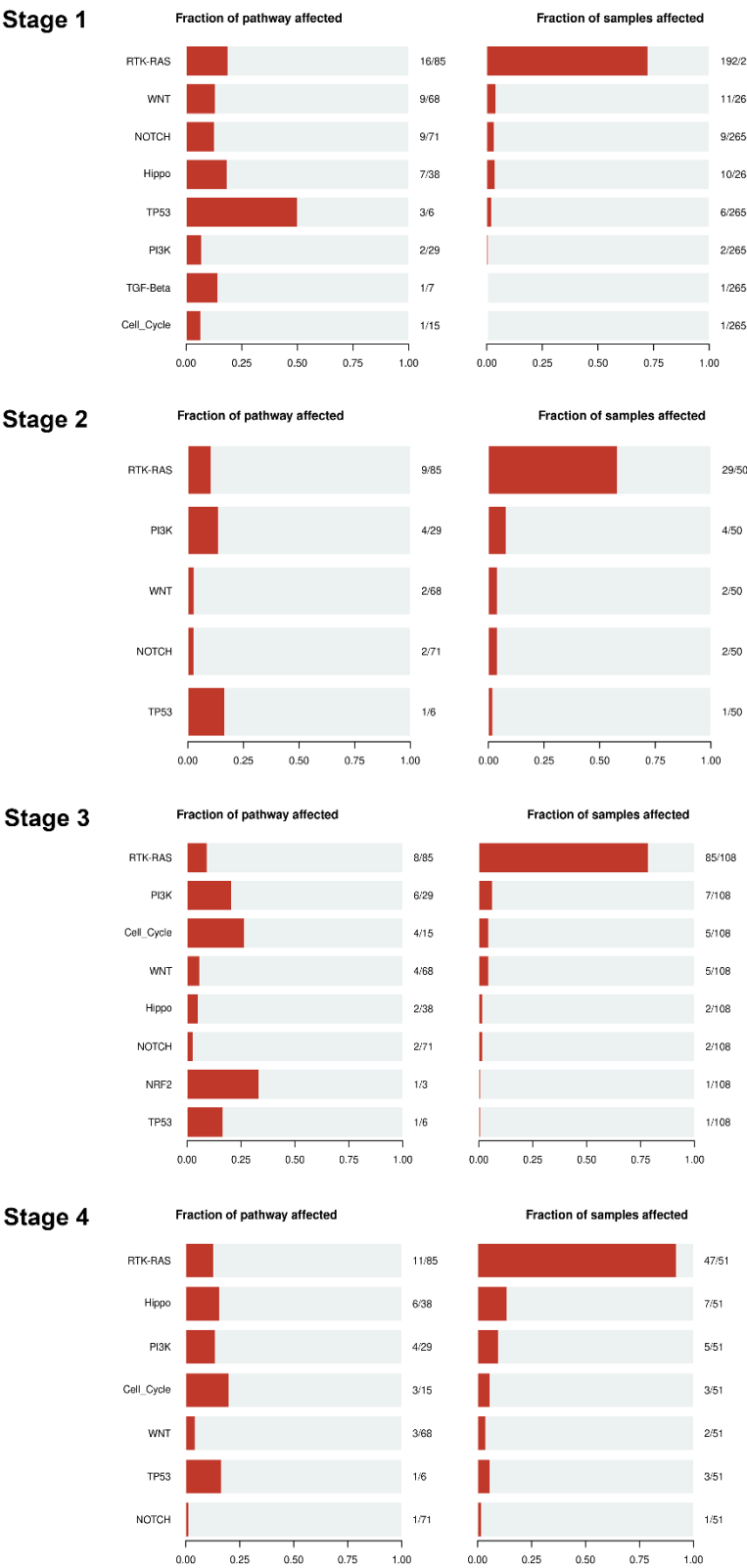

Supplement: Supplementary file 6 — Additional file 6: Figure S1. Altered pathways in different tumor stages in PTC. [file 12935_2022_2680_MOESM6_ESM.pdf]
